# Supplementary material for: Structure guided studies of the interaction between PTP1B and JAK
Source: Commun Biol. 2023 Jun 14;6:641. doi: 10.1038/s42003-023-05020-9 (PMC10267100; doi:10.1038/s42003-023-05020-9)
Supplement: Supplementary file 2 — Supplementary Information [file 42003_2023_5020_MOESM2_ESM.pdf]

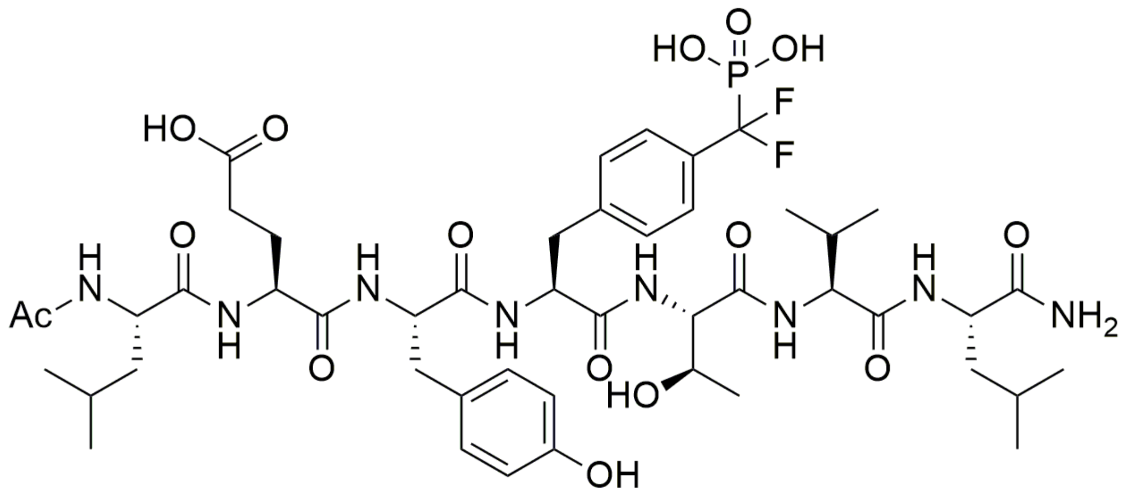


**Supplementary Figure S1. Structure of the non-hydrolyzable JAK1 DiF peptide used for thermal shift experiments.** PTP1B is unable to dephosphorylate the diF phosphotyrosine analogue.

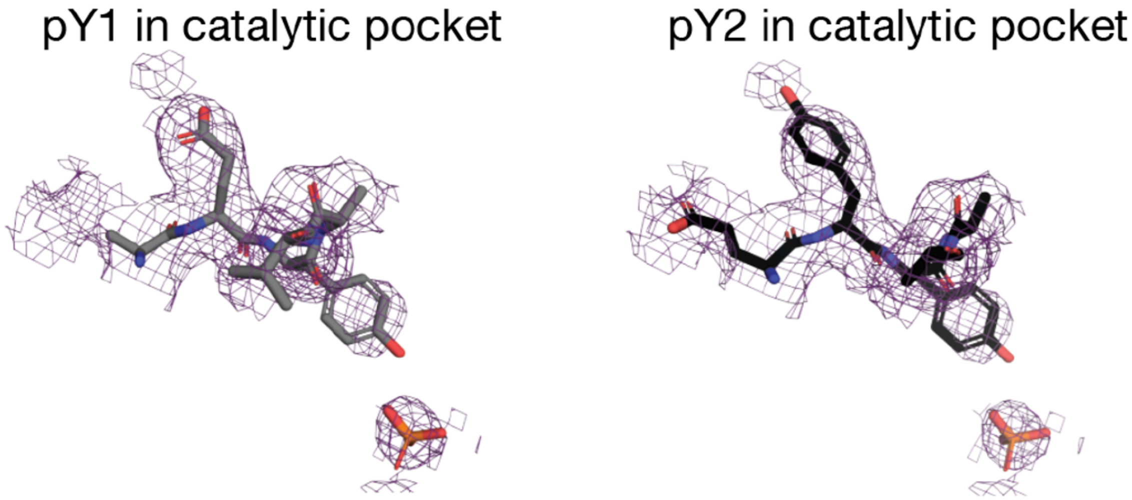


**Supplementary Figure S2. Potentially scrambled peptide interactions with JAK and PTP1B DQ.** 2Fo-Fc composite omit map with simulated annealing, contoured to 1σ for the JAK2 pYpY activation loop peptide with first or second tyrosine in catalytic pocket.

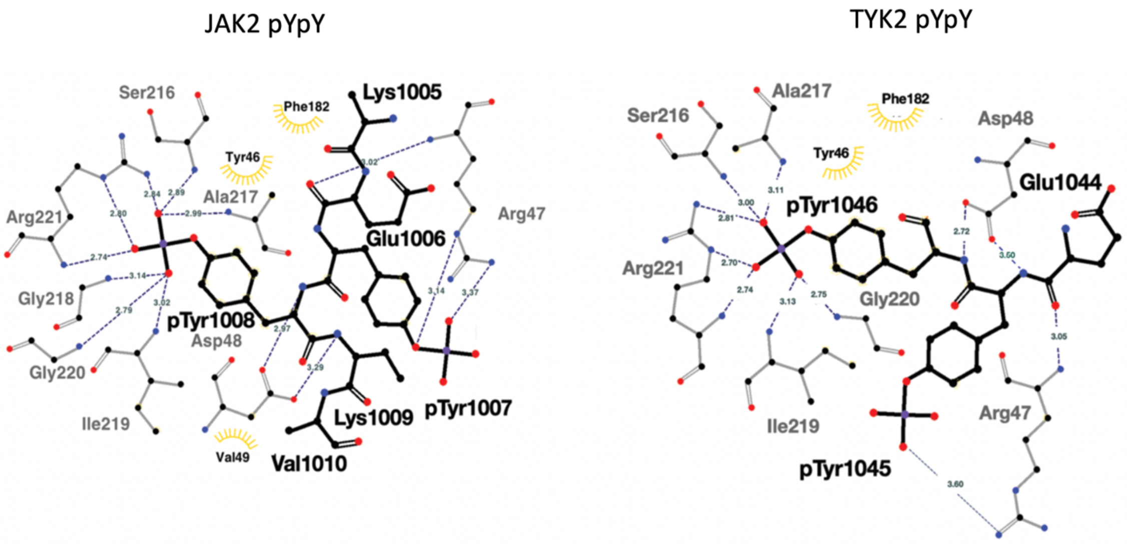


**Supplementary Figure S3. Interactions between PTP1B DQC in complex with JAK2 and TYK2 activation loop peptides.** 2D representation of the interactions between PTP1B and the TYK2 activation loop peptide. For both 2D representations, interactions are indicated by dashed lines, with the distance between the residues in angstroms indicated. Dotted lines represent hydrogen bonds or electrostatic interactions. Yellow dashes crescents represent hydrophobic interactions. Visualized using LigPlot+ (Laskowski and Swindells 2011).

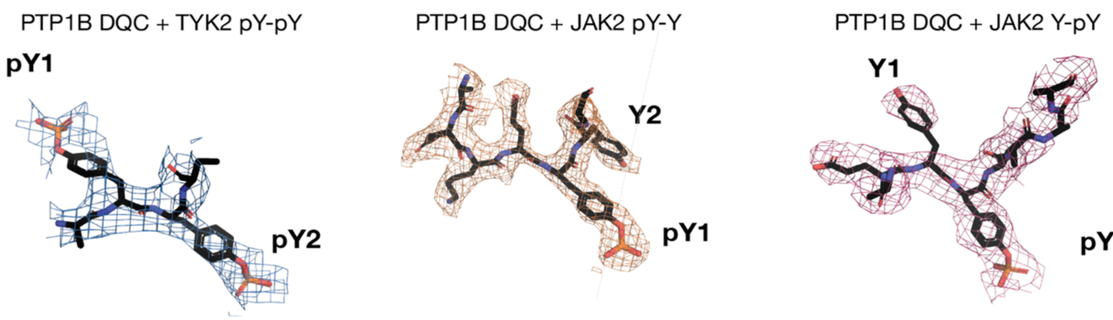


**Supplementary Figure S4. Omit maps for phosphopeptides bound to the PTP1B.** 2Fo-Fc composite omit map with simulated annealing, contoured to 0.8 σ for the TYK2 pY-pY phosphopeptide bound to PTP1B DQC, the JAK2 pY-Y phosphopeptide bound to PTP1B DQC, and the JAK2 Y-pY phosphopeptide bound to PTP1B DQC.

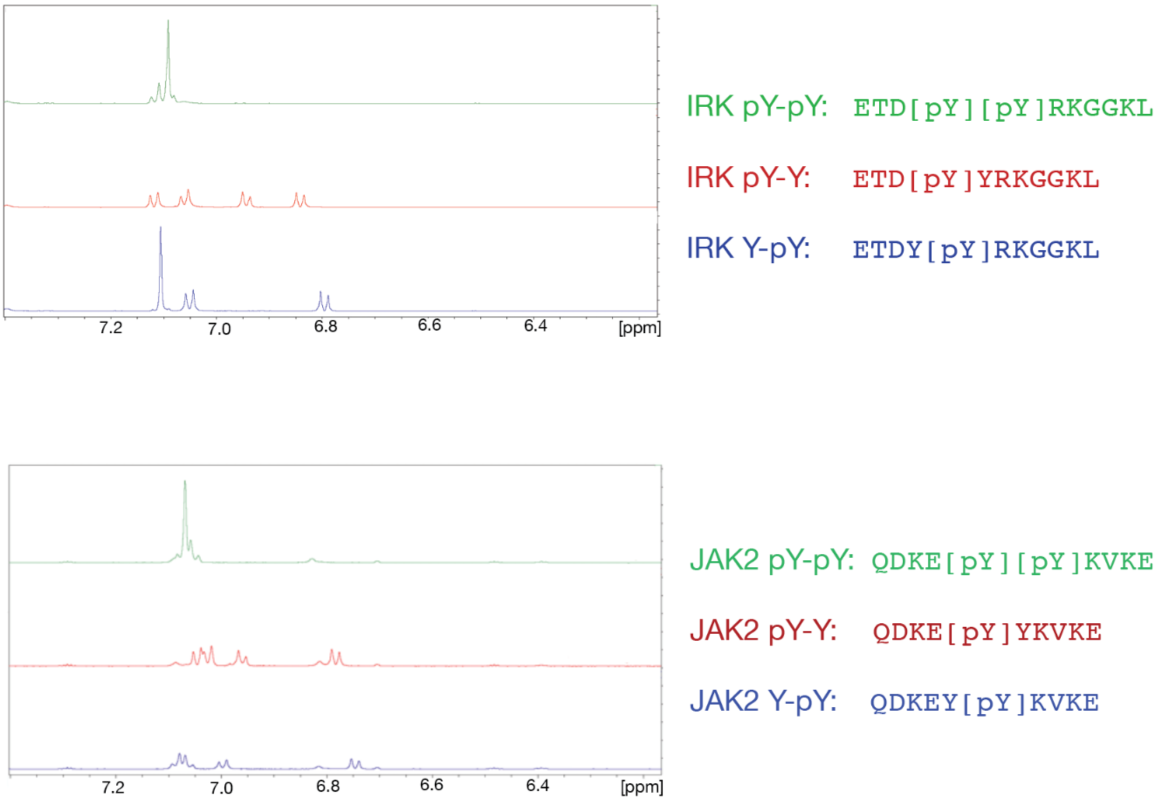


**Supplementary Figure S5. 1D 1H solution NMR spectra.** JAK2 pYpY (green) peaks measured between 7.03-7.10 ppm, pYY (red) peaks measured 6.93- 6.97 and 6.97-7.01 ppm and YpY (blue) peaks measured between 7.01-6.97 ppm, and sequences of each peptide. Spectra of tyrosine residues for IRK pYpY (green) peaks measured between 7.07-7.11 ppm, pYY (red) peaks measured between 6.92- 6.96 ppm and YpY (blue) peaks measured between 6.78- 6.81 ppm and sequences of each peptide.

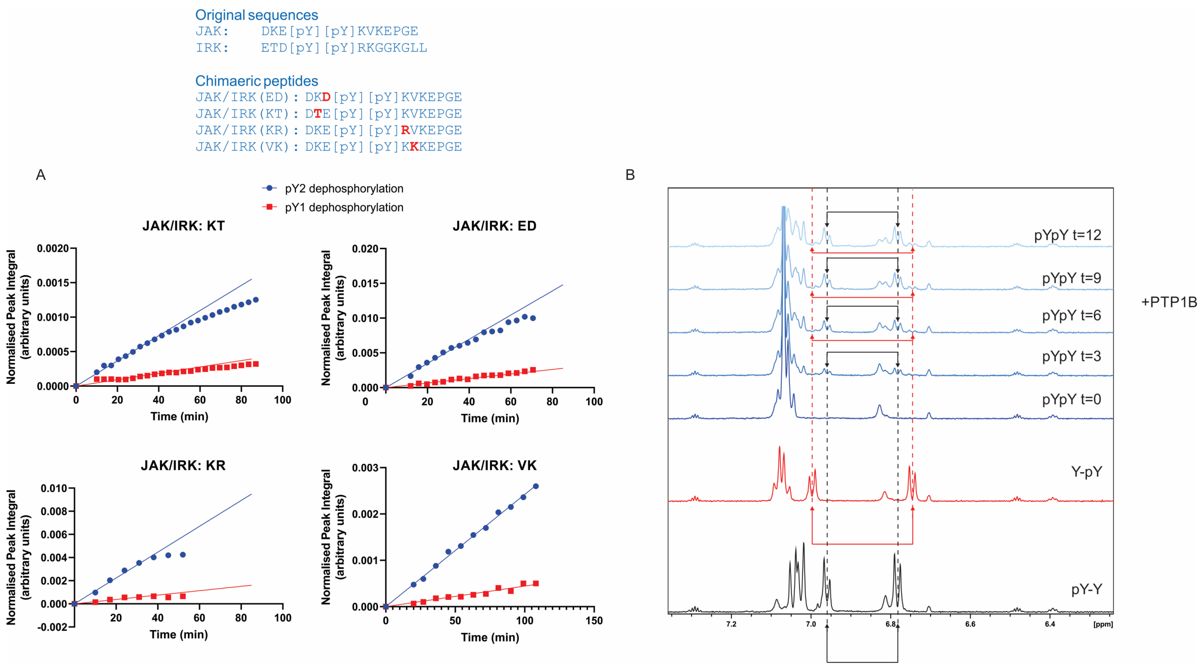


**Supplementary Figure S6 Dephosphorylation of JAK2 peptides with IRK residues.** Dephosphorylation of JAK2/IRK chimaeric peptides by PTP1B as tracked by $^1$H NMR. (A) Four dephosphorylated JAK/IRK chimaeric peptides were synthesized that each contained a single altered residue at the -2 to -2 positions (relative to pTyr-pTyr). Dephosphorylation of each of these by PTP1B was tracked over time using $^1$H NMR. (B) An example of tracking the progress of PTP1B-induced dephosphorylation. Each of the singly-phosphorylated peptides has at least one peak doublet (the delta or epsilon protons on the Tyr ring, split via J-coupling to the neighbouring proton) that can be used as a fingerprint for appearance of that species during the course of the reaction (shown in red and black). After addition of PTP1B to the bis-phsophorylated species (blue traces) peaks corresponding to the pY-Y species appear 4-6x more rapidly (as quantified via peak integration) than peaks corresponding to Y-pY.

**Supplementary Figure S7:** Raw uncropped western blots alongside the cropped versions to show which bands were used.

Exp 1

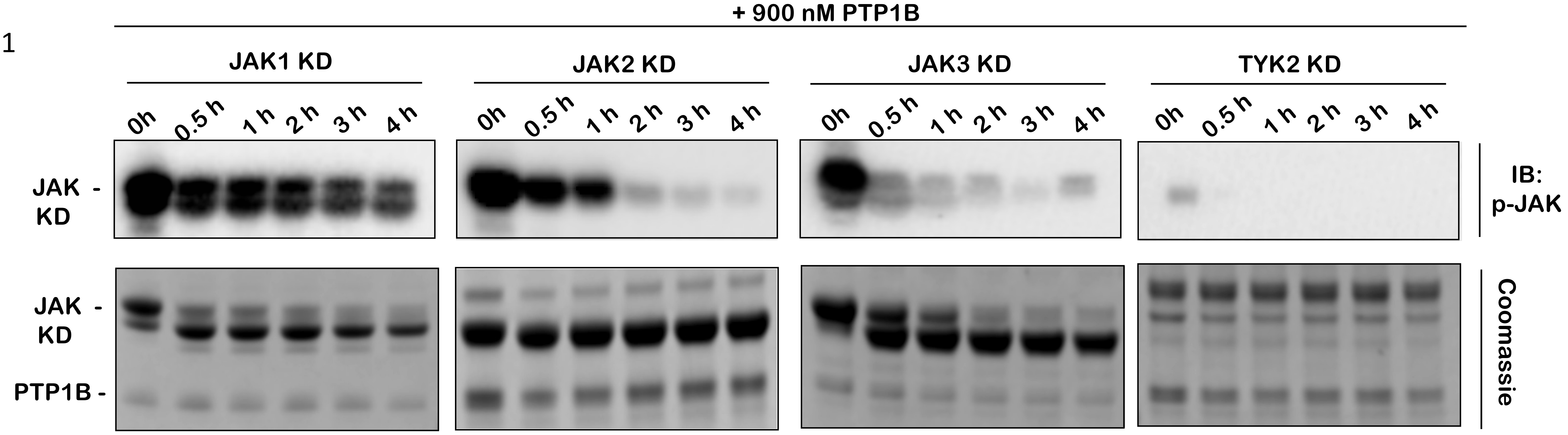


Raw p-JAK (JAK1, JAK2, JAK3, TYK2)

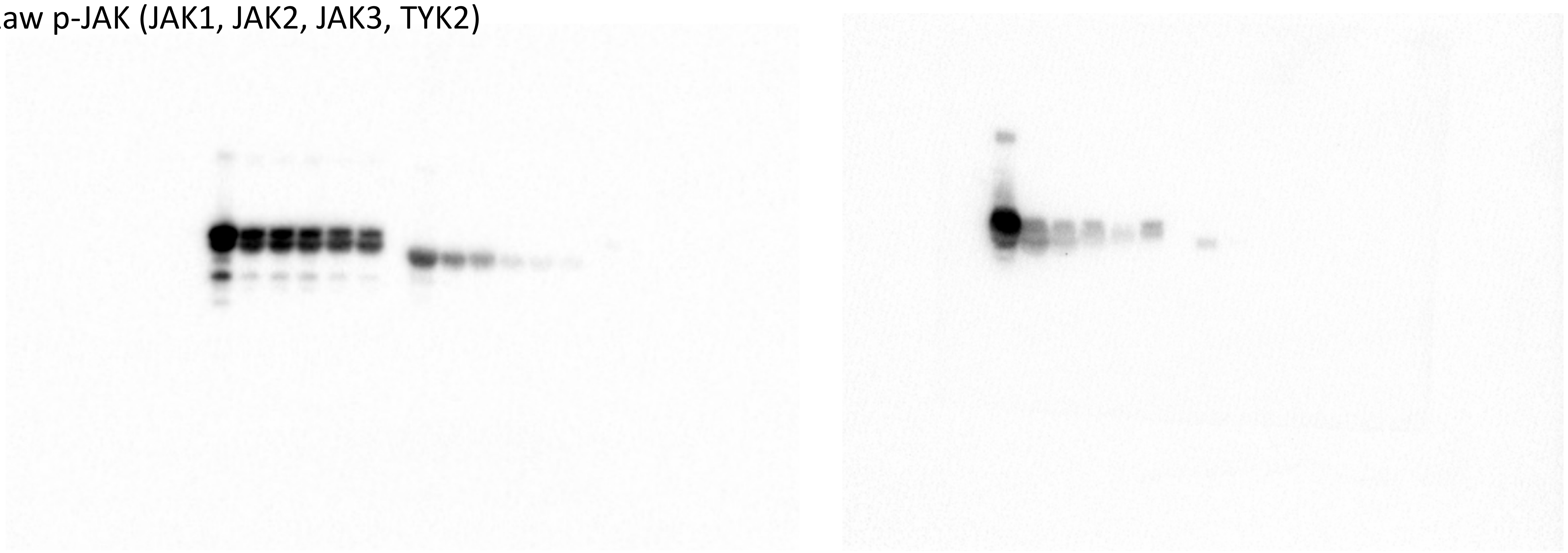

Raw Coomassie (JAK1, JAK2, JAK3, TYK2)

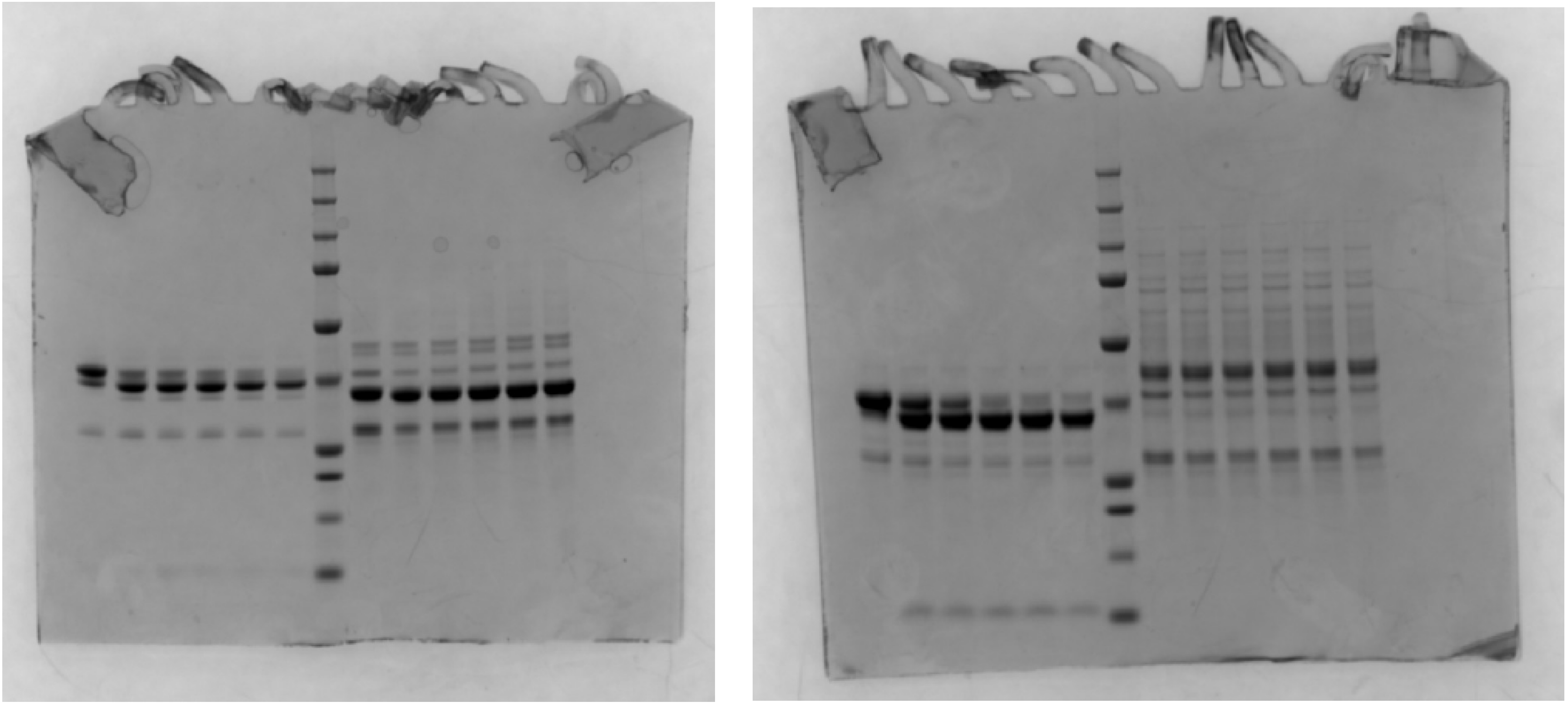

Exp 2

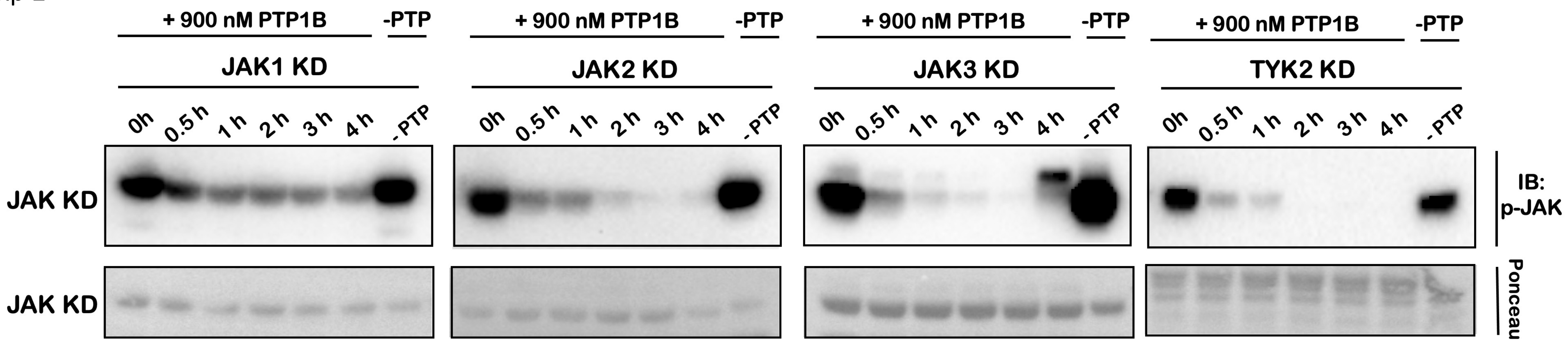


Raw Coomassie (JAK1, JAK2, JAK3, TYK2)

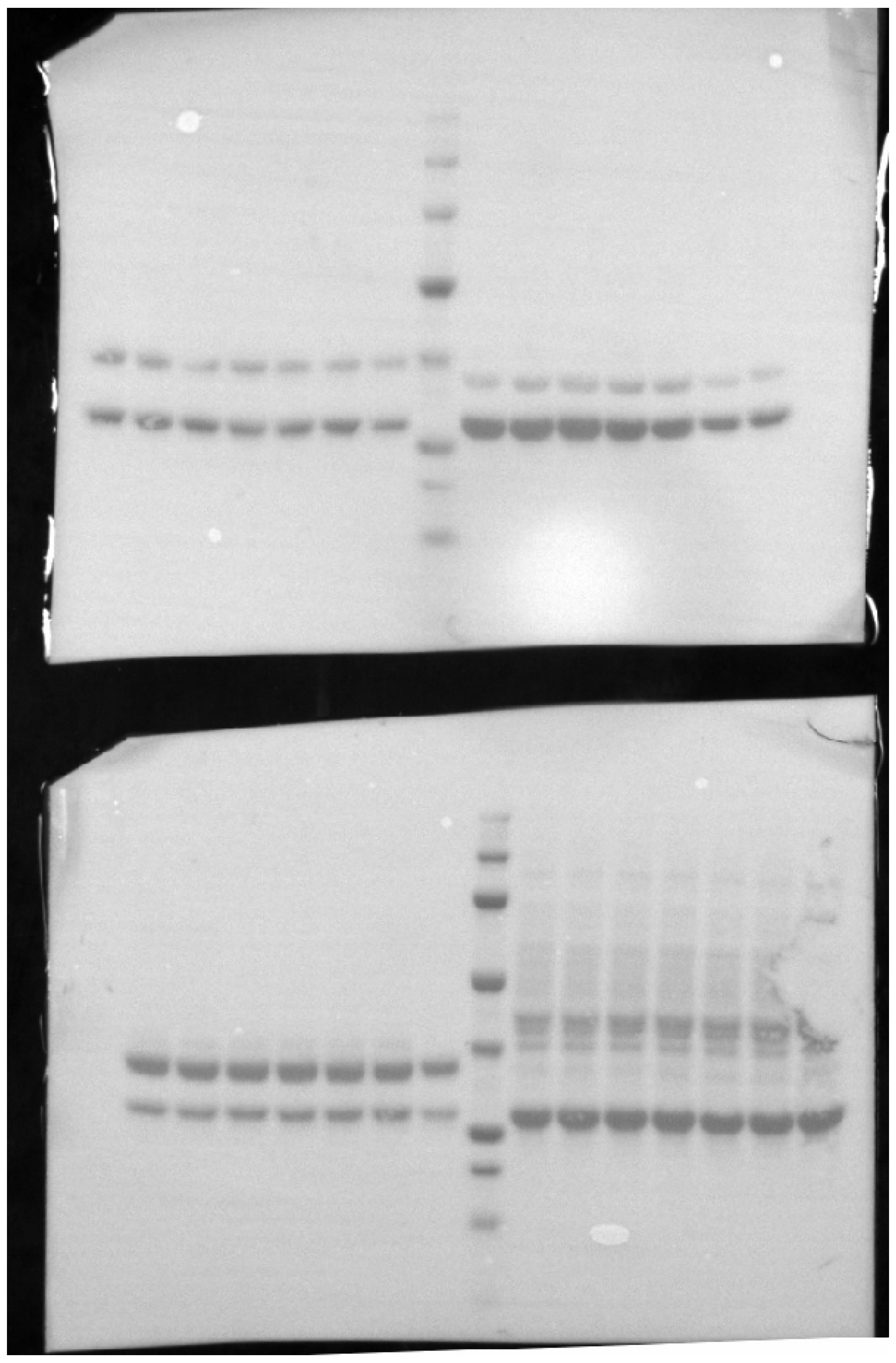

Raw p-JAK (JAK1, JAK2, JAK3, TYK2)
Short exposure

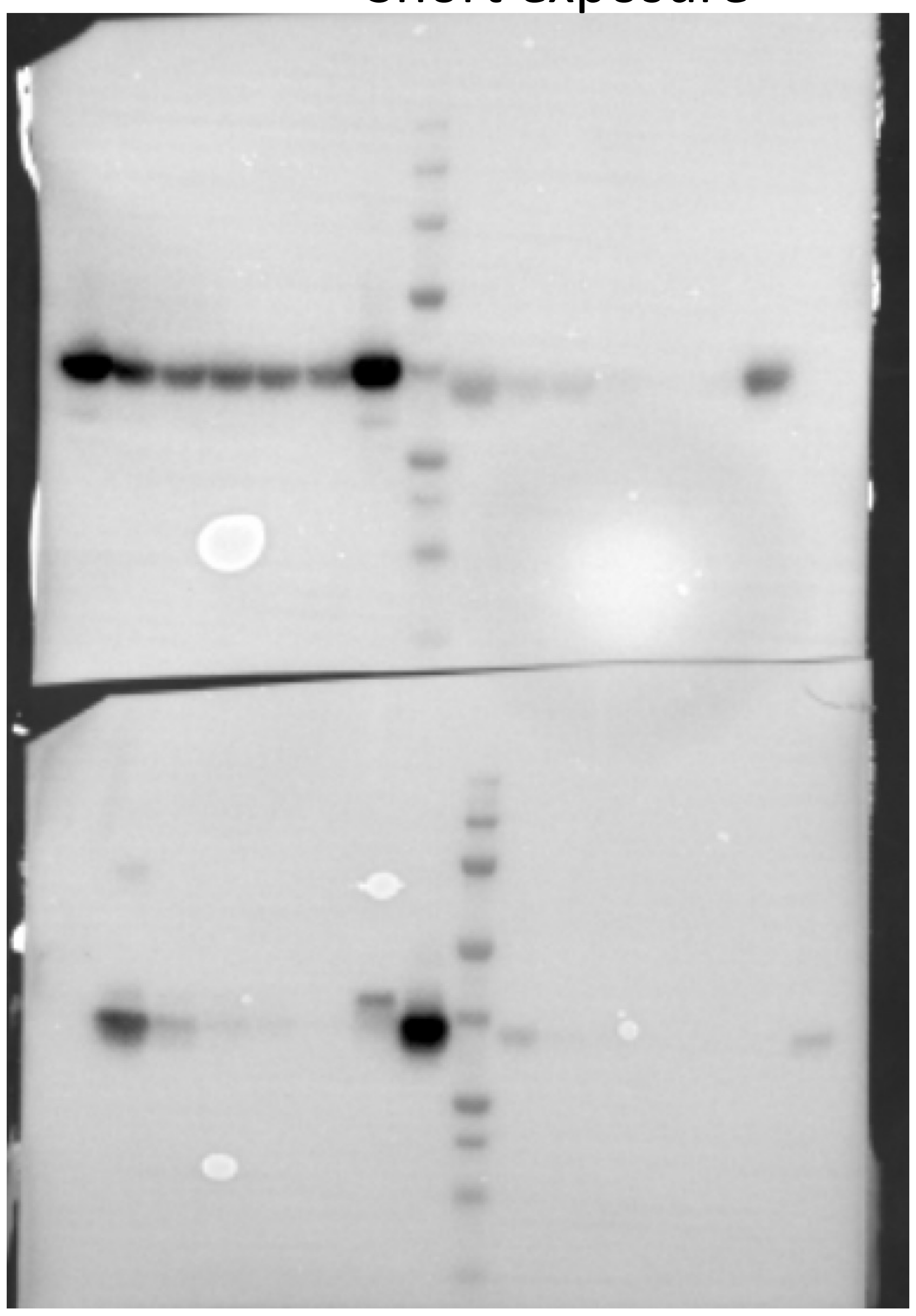

Raw p-JAK (JAK1, JAK2, JAK3, TYK2)
Long exposure

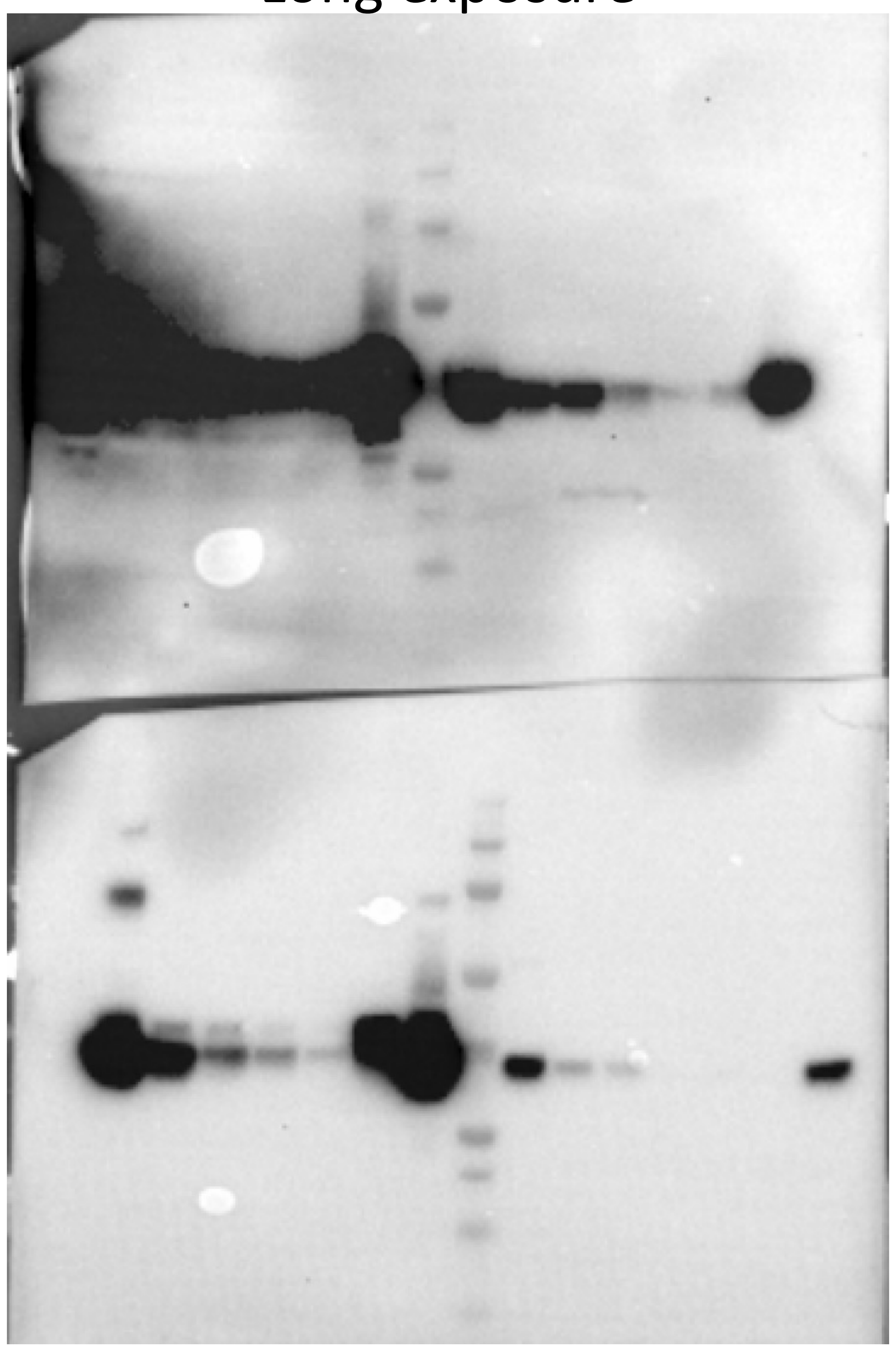

Exp 3

Raw p-JAK
(JAK1, JAK2)

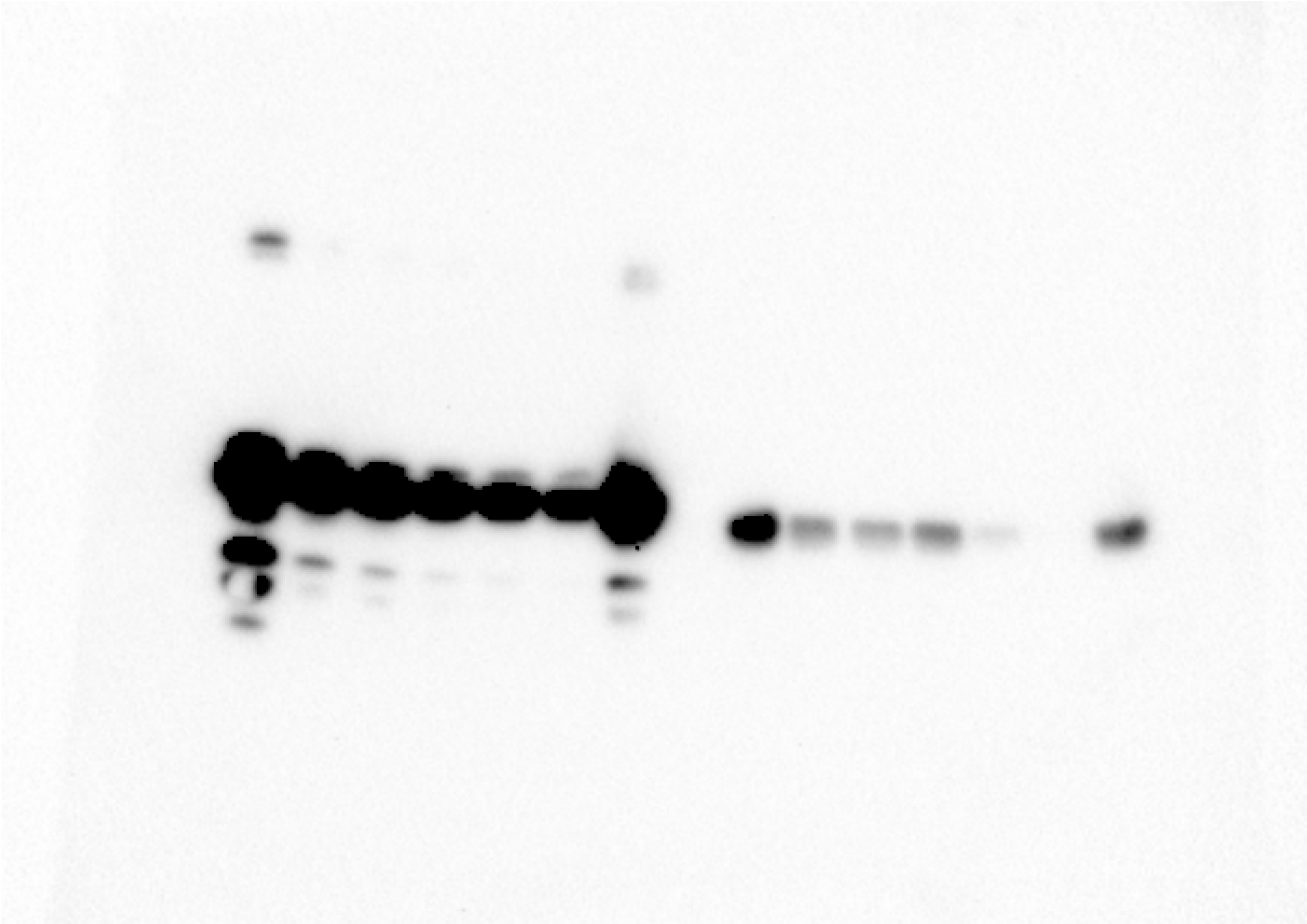

Raw p-JAK
(JAK3, TYK2)

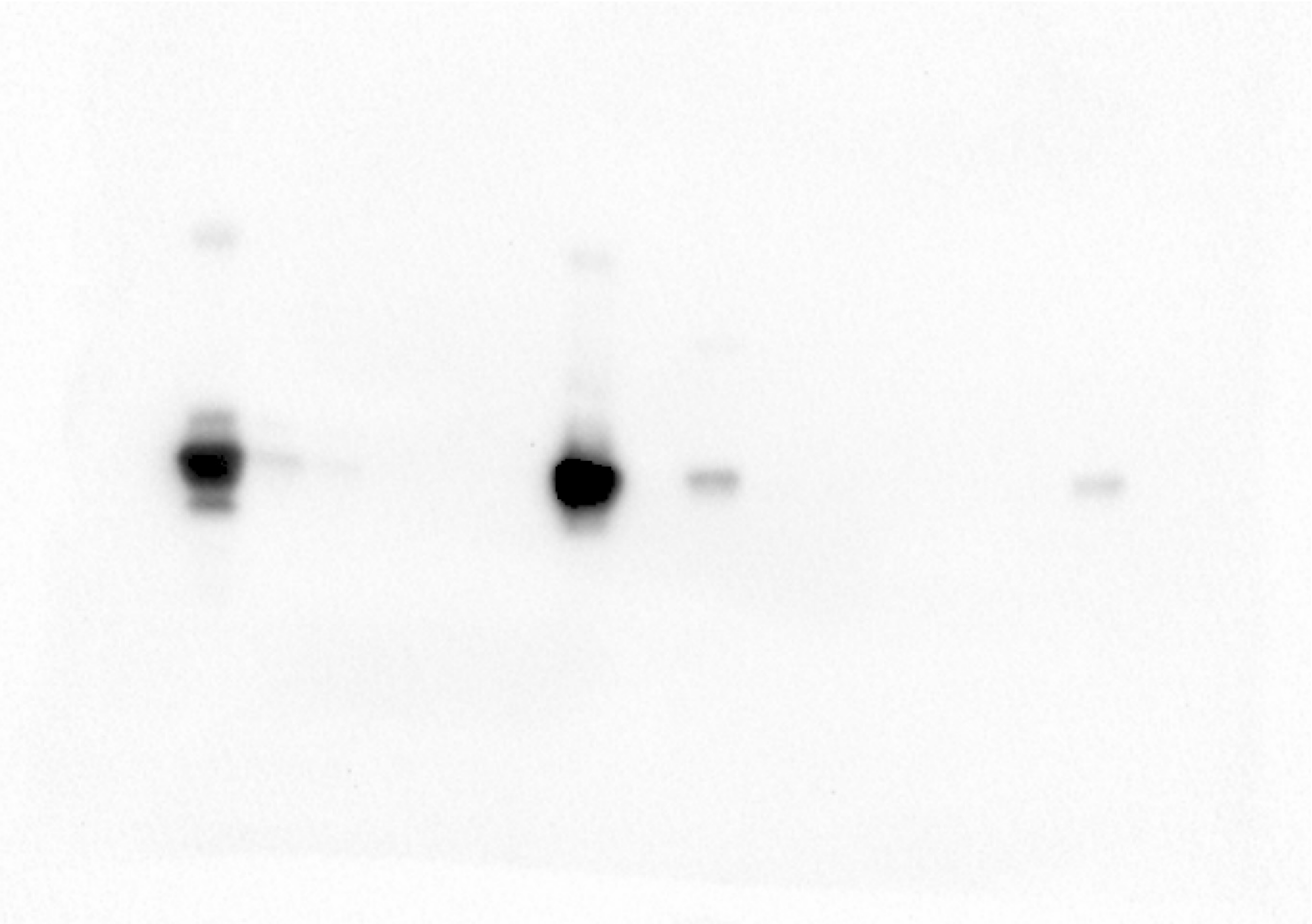

Exp 4

PTP1B

Raw p-JAK
(JAK1, JAK2)

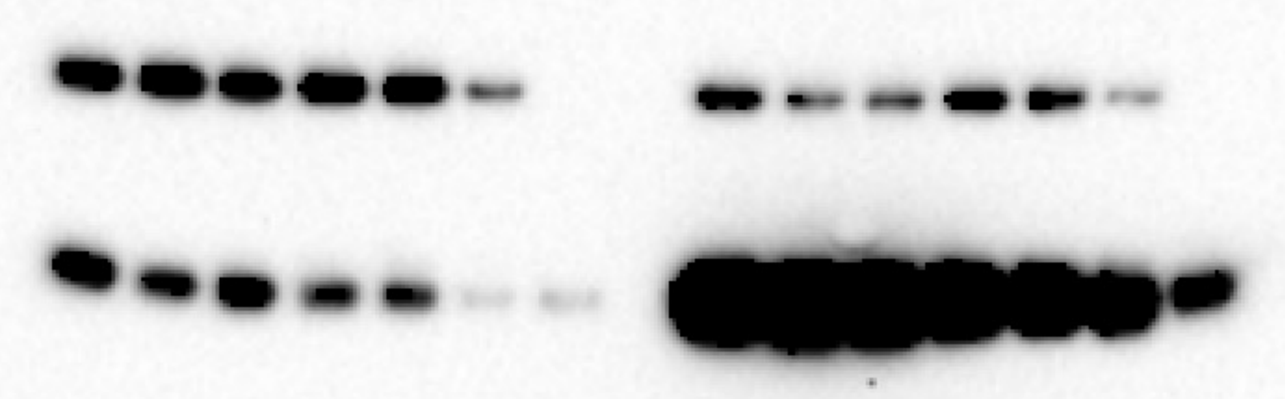

PTP1B

Raw p-JAK
(JAK3, TYK2)

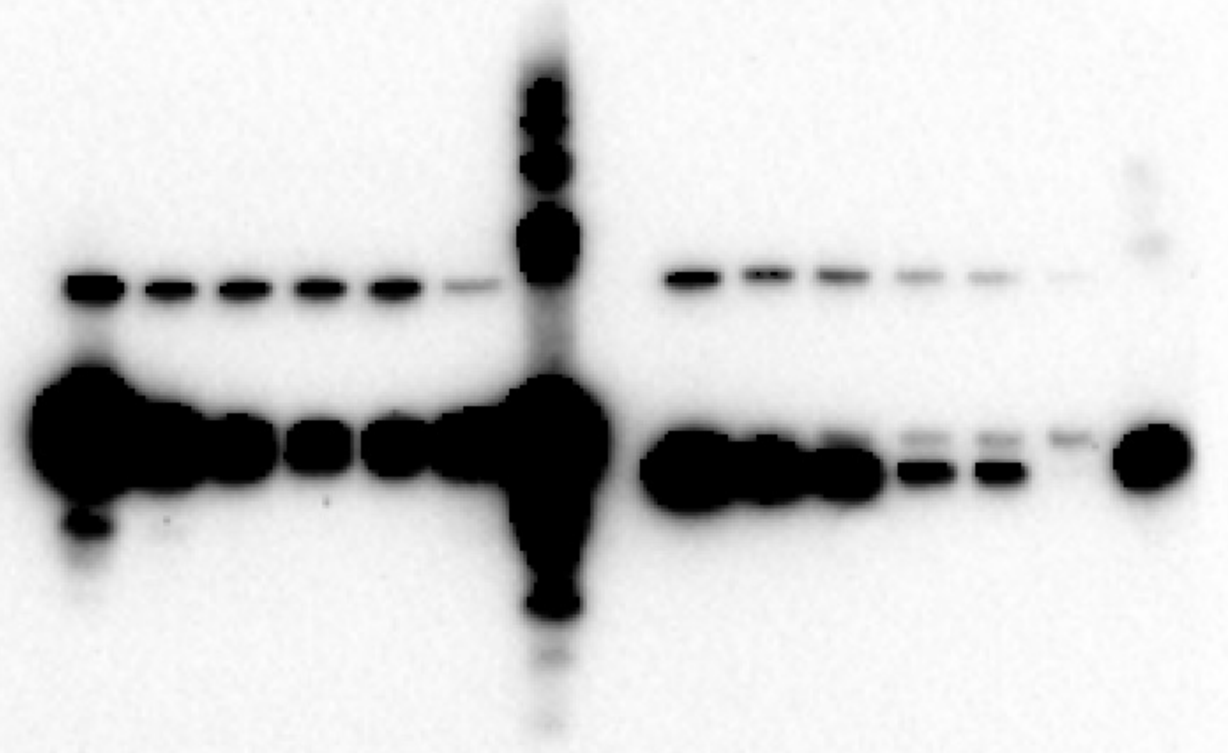

Exp 5

Raw p-JAK
(JAK3)

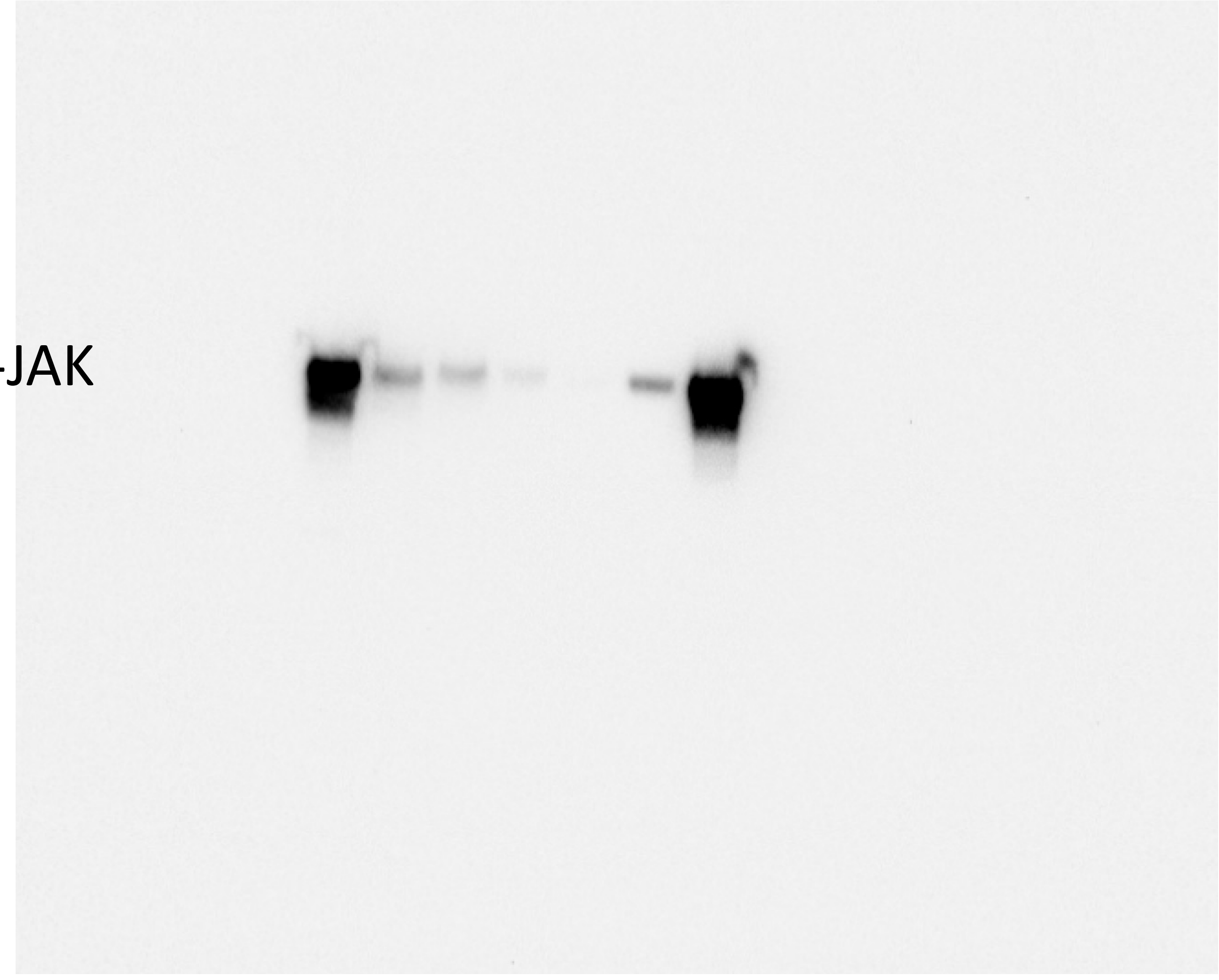

Raw p-JAK
(JAK1, JAK2,
TYK2)

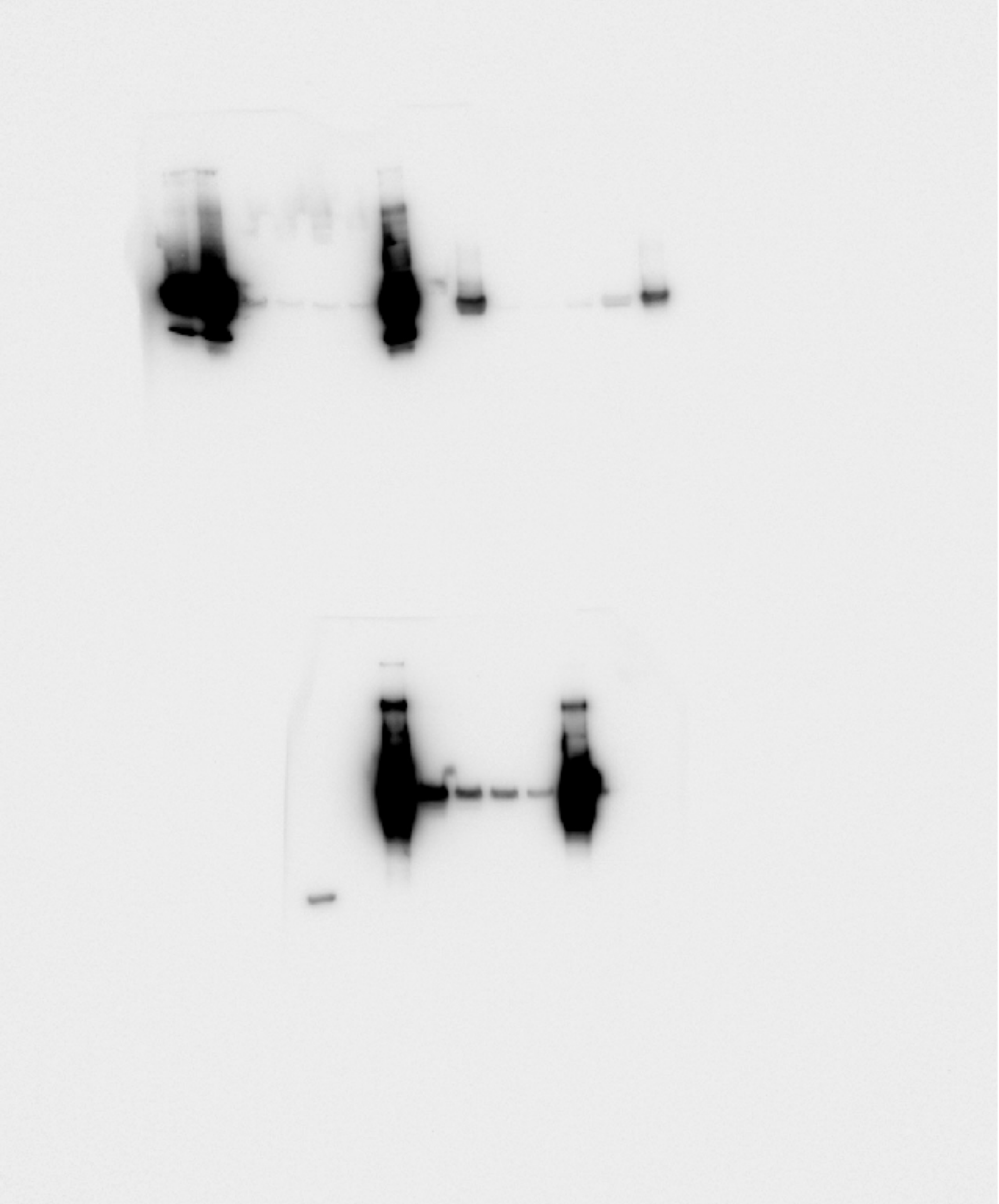

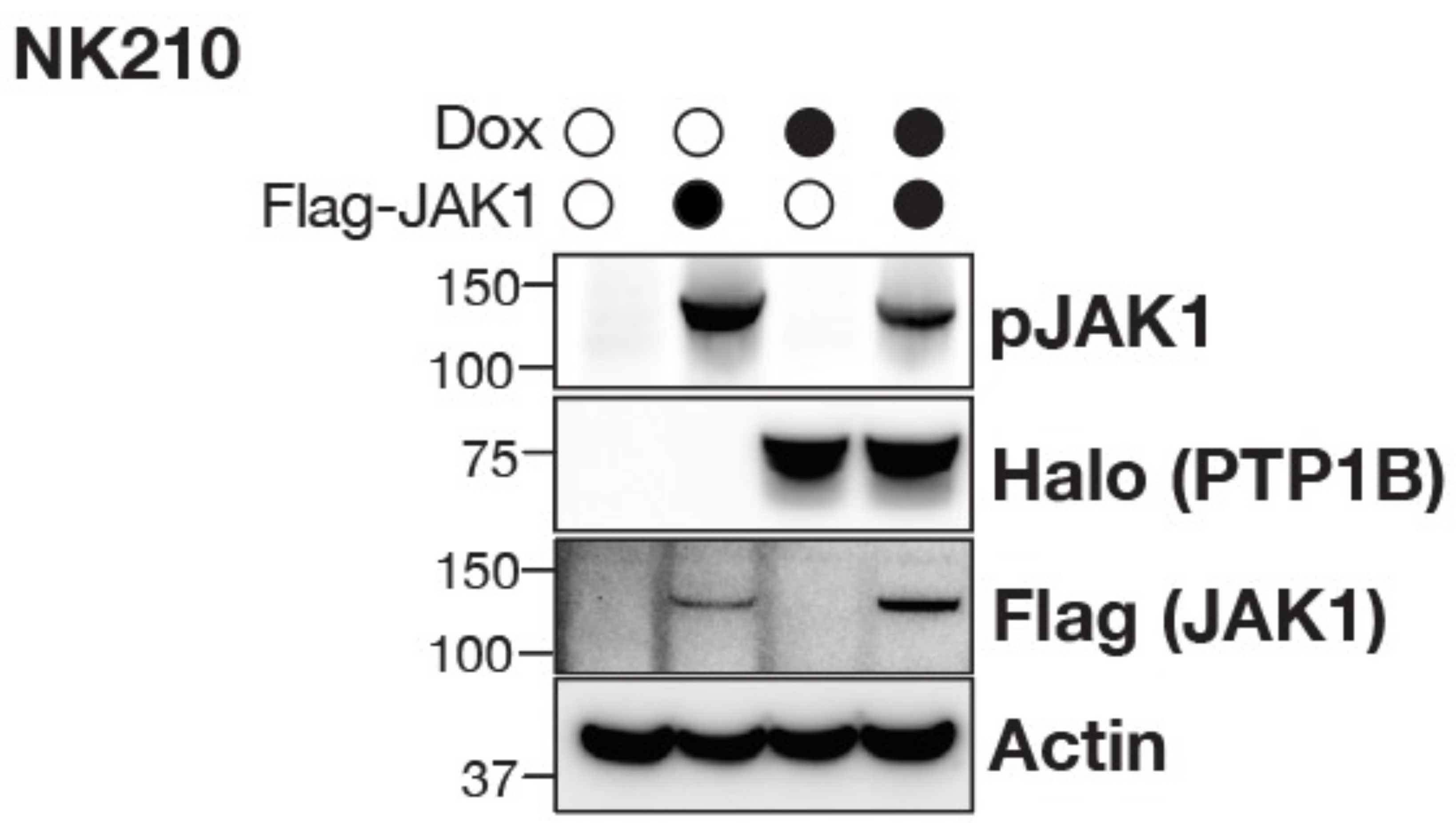


FLAG-JAK

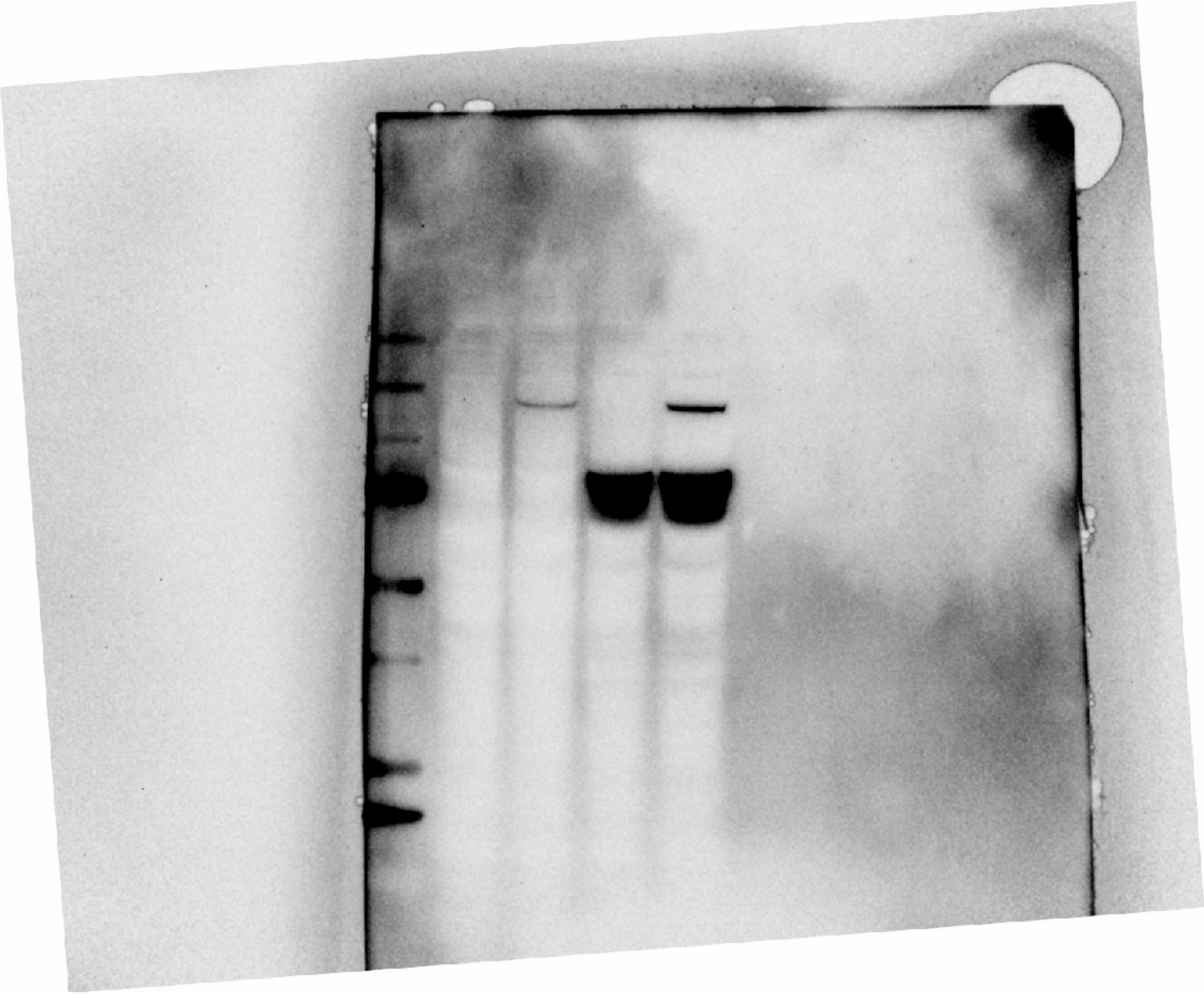

Actin

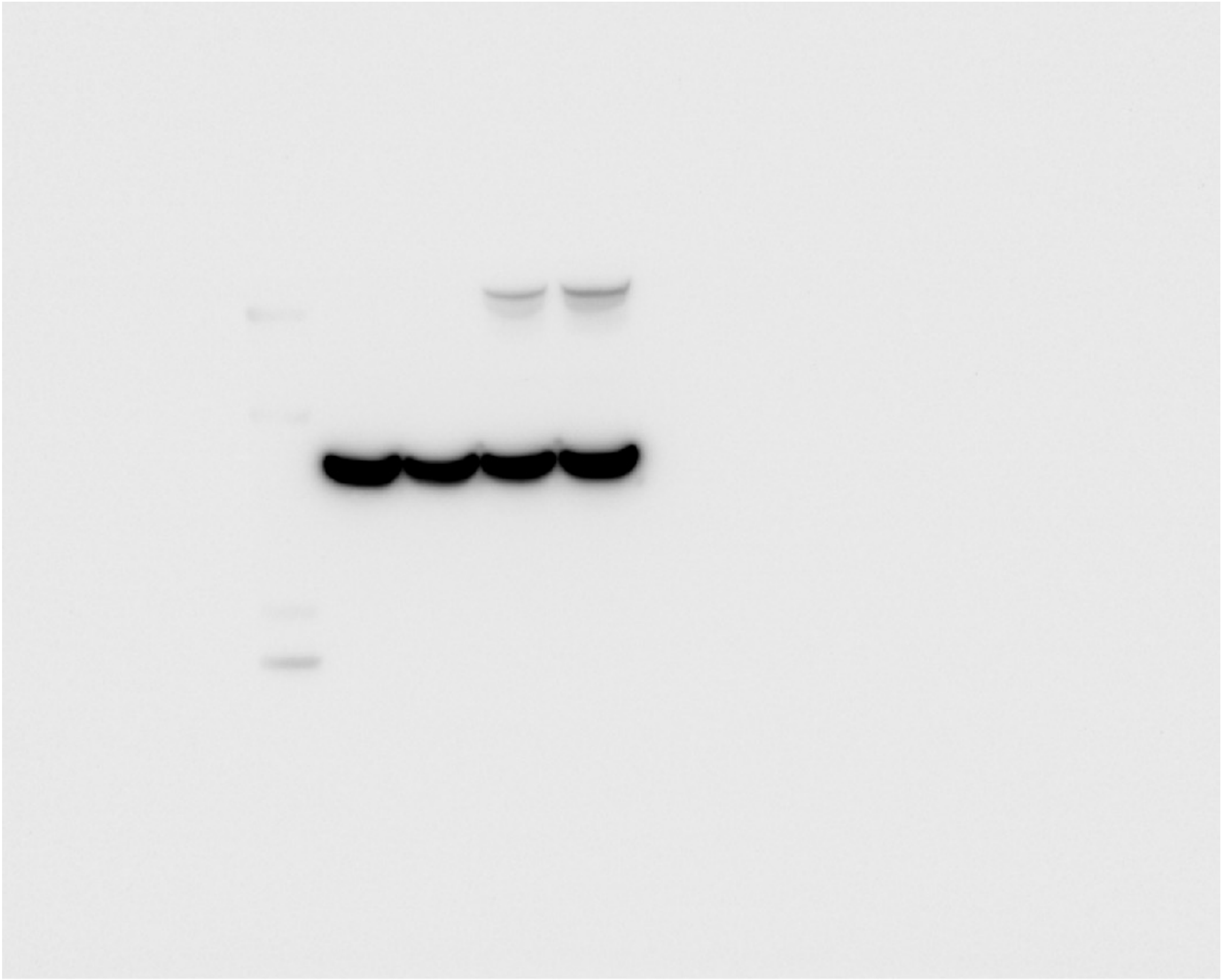

Halo-PTP1B

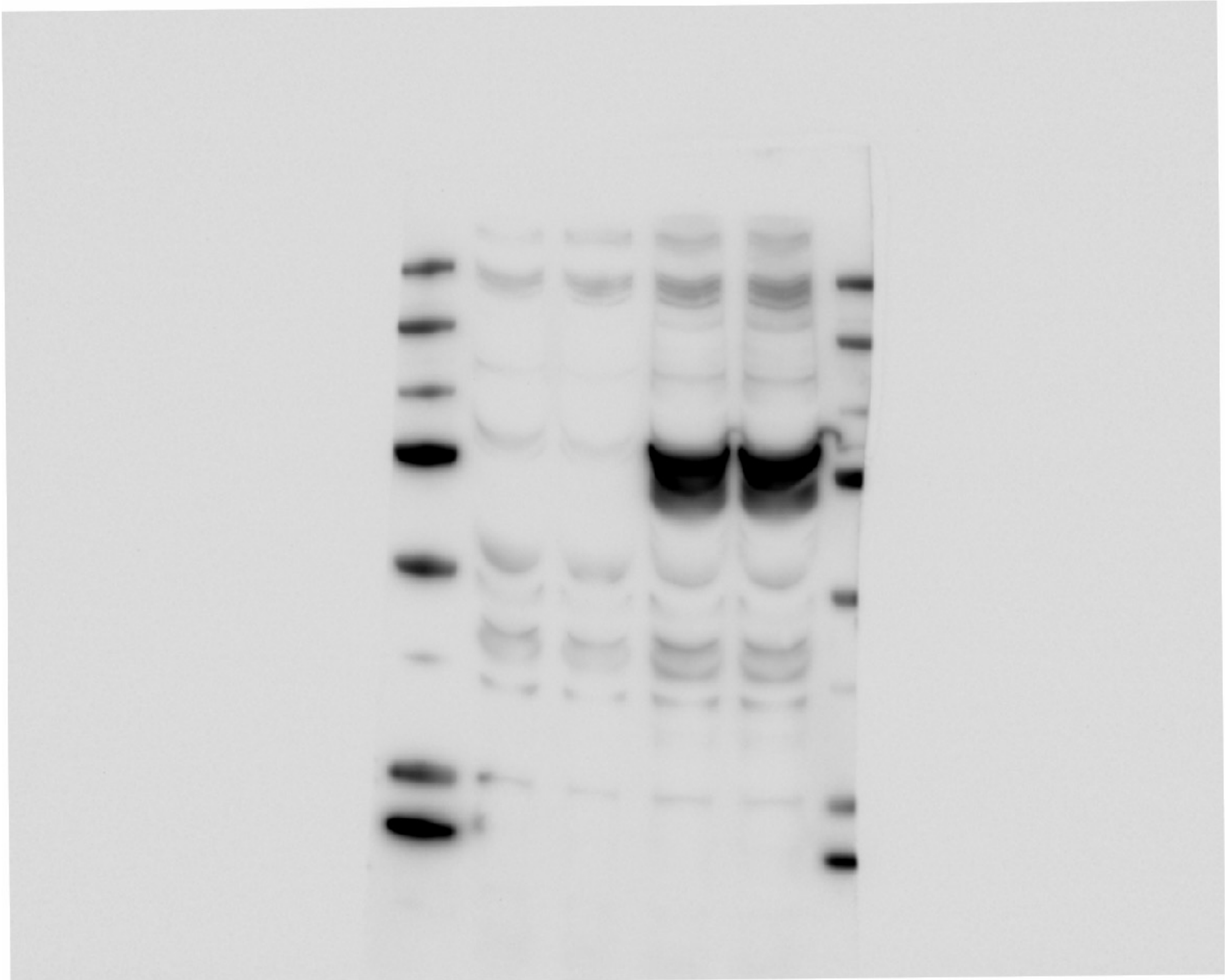

pJAK

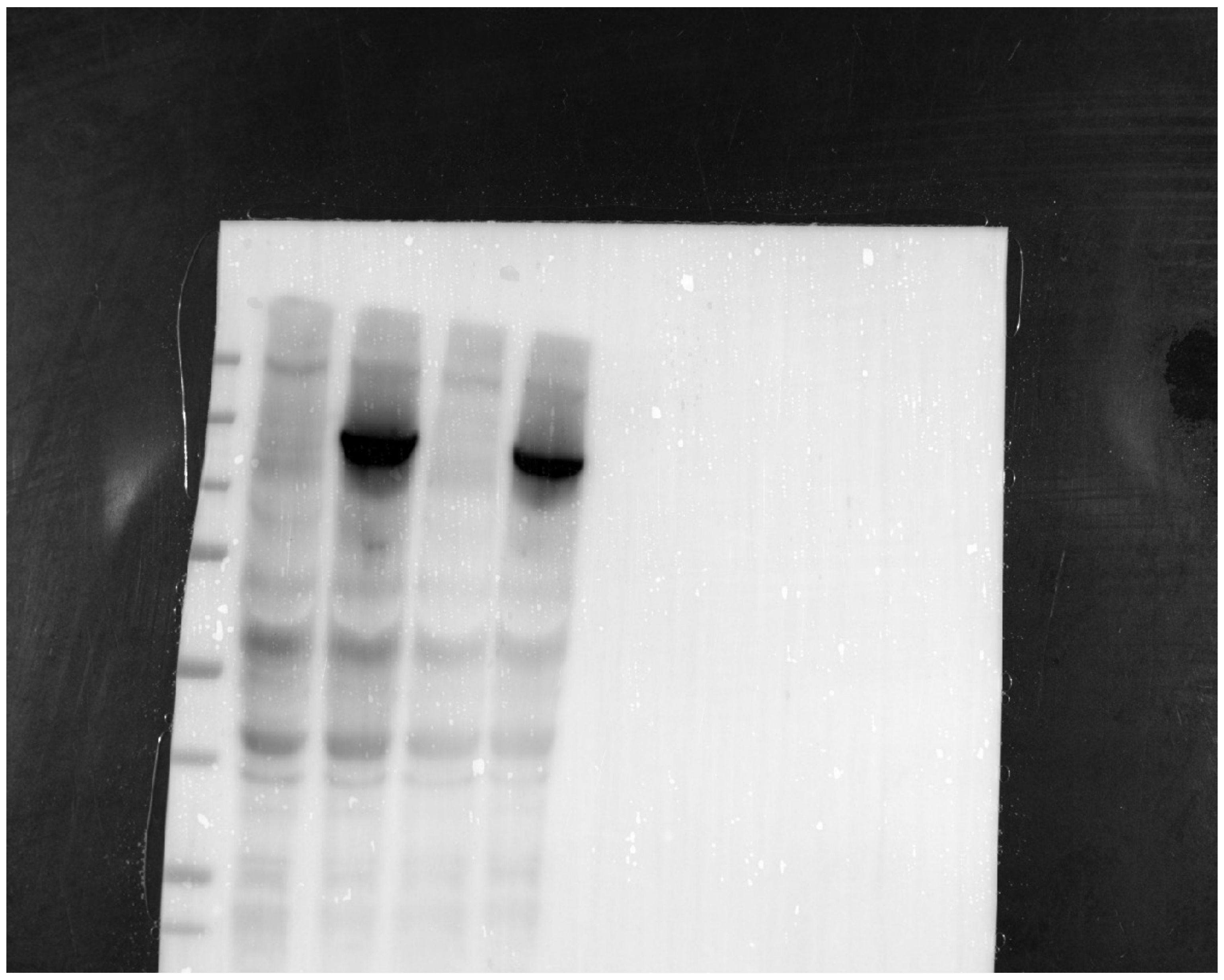

**NK214**

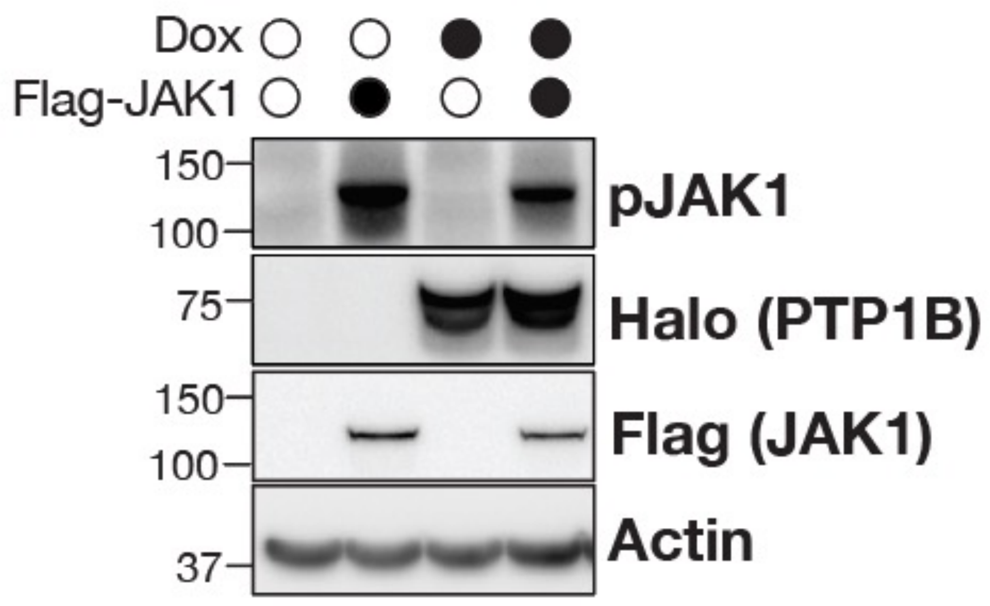
Dox
Flag-JAK1
150
100
pJAK1
75
Halo (PTP1B)
150
100
Flag (JAK1)
37
Actin

**NK215**

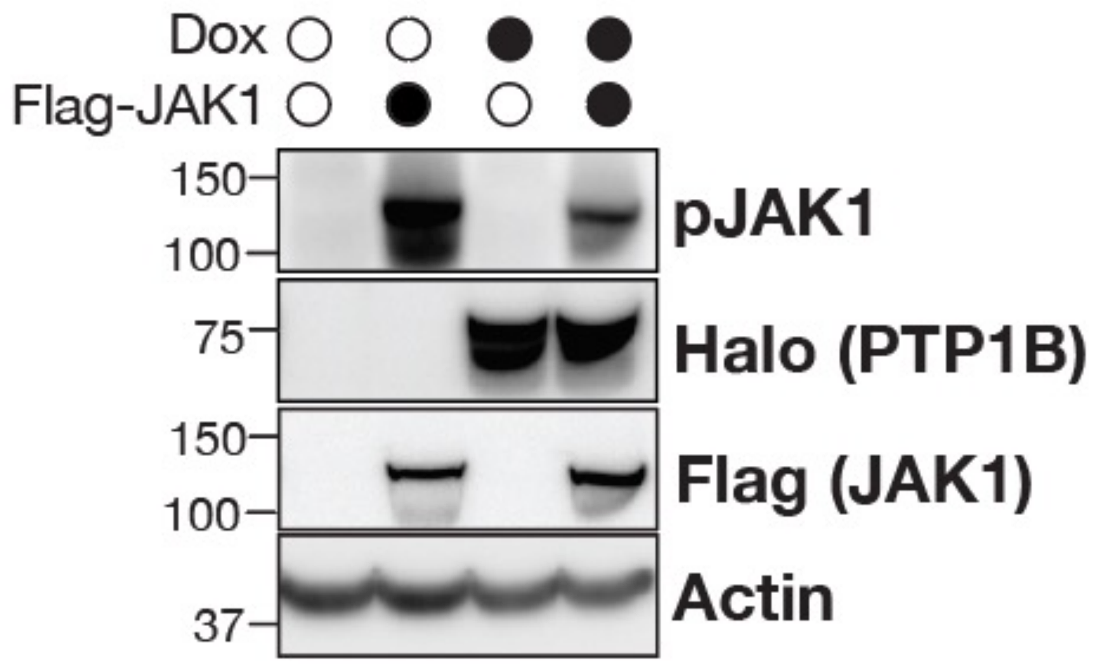
Dox
Flag-JAK1
150
100
pJAK1
75
Halo (PTP1B)
150
100
Flag (JAK1)
37
Actin

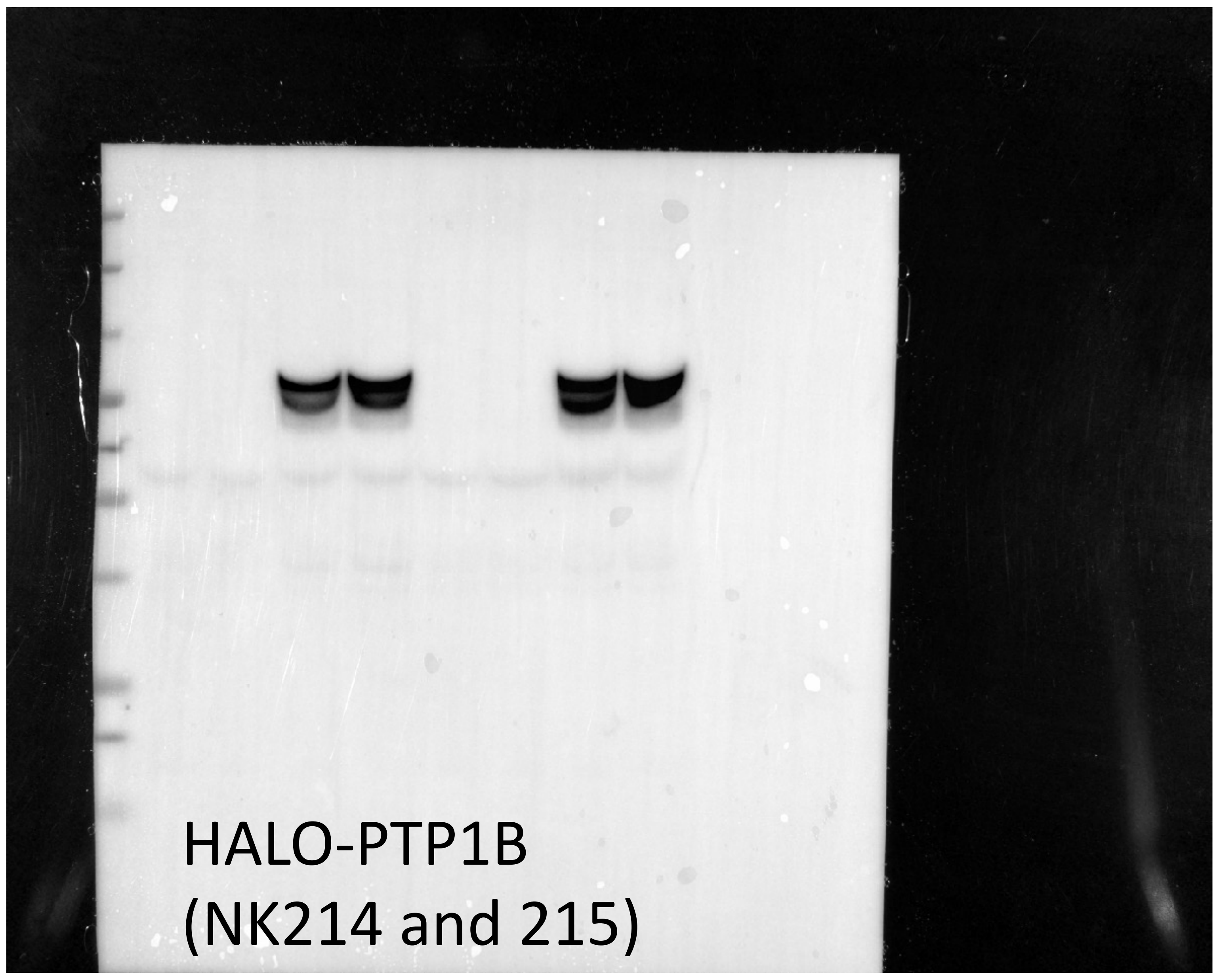
HALO-PTP1B
(NK214 and 215)

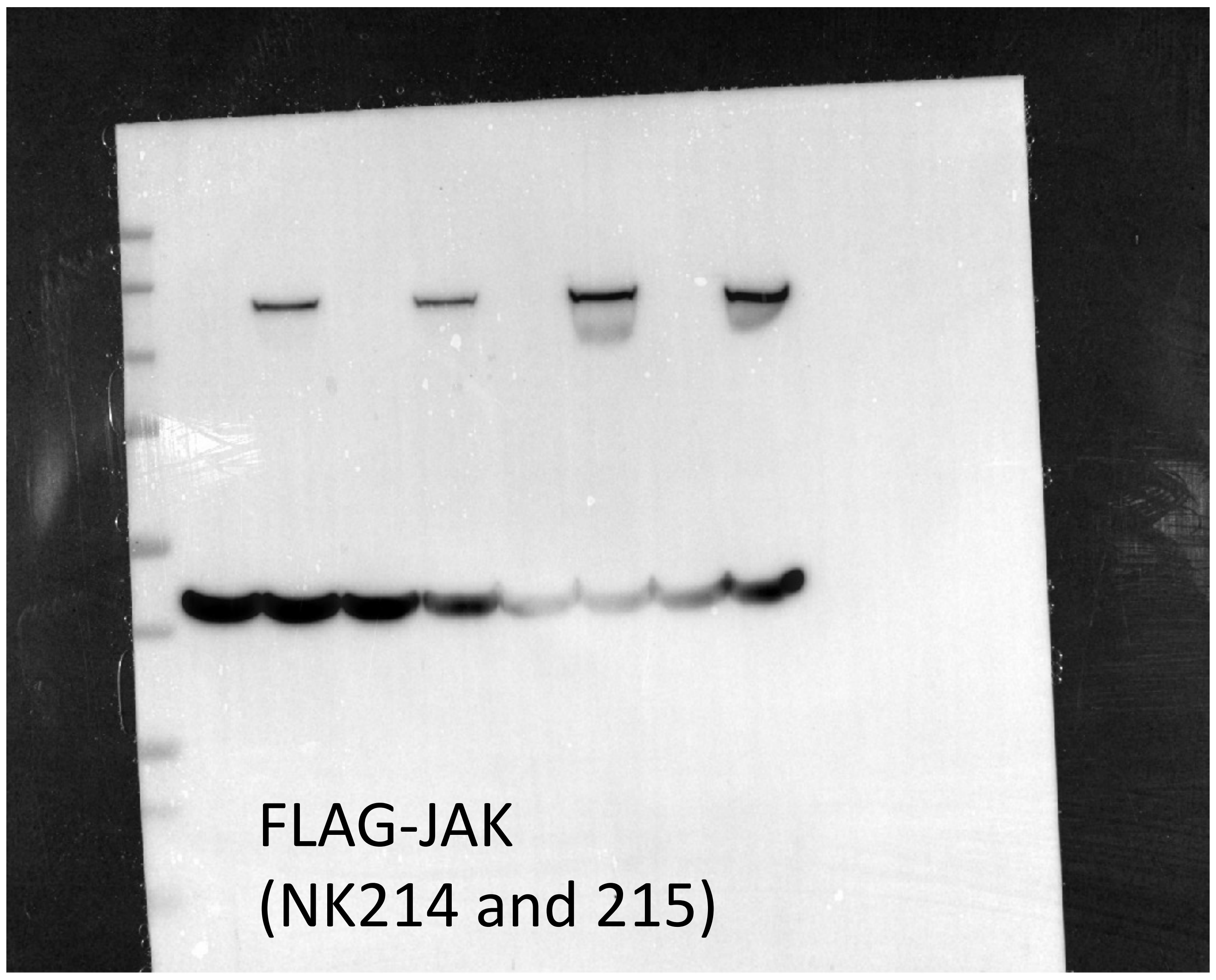
FLAG-JAK
(NK214 and 215)

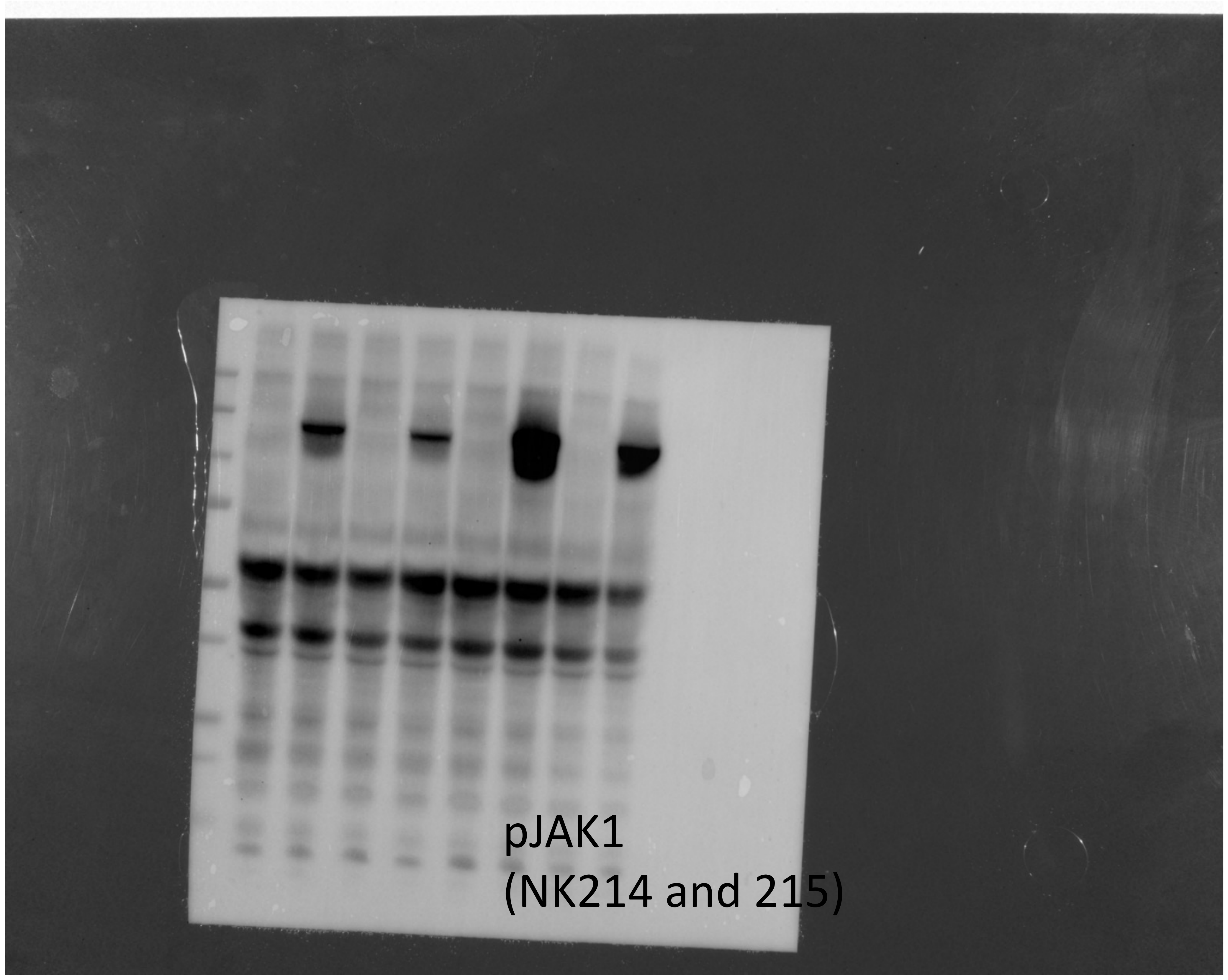
pJAK1
(NK214 and 215)

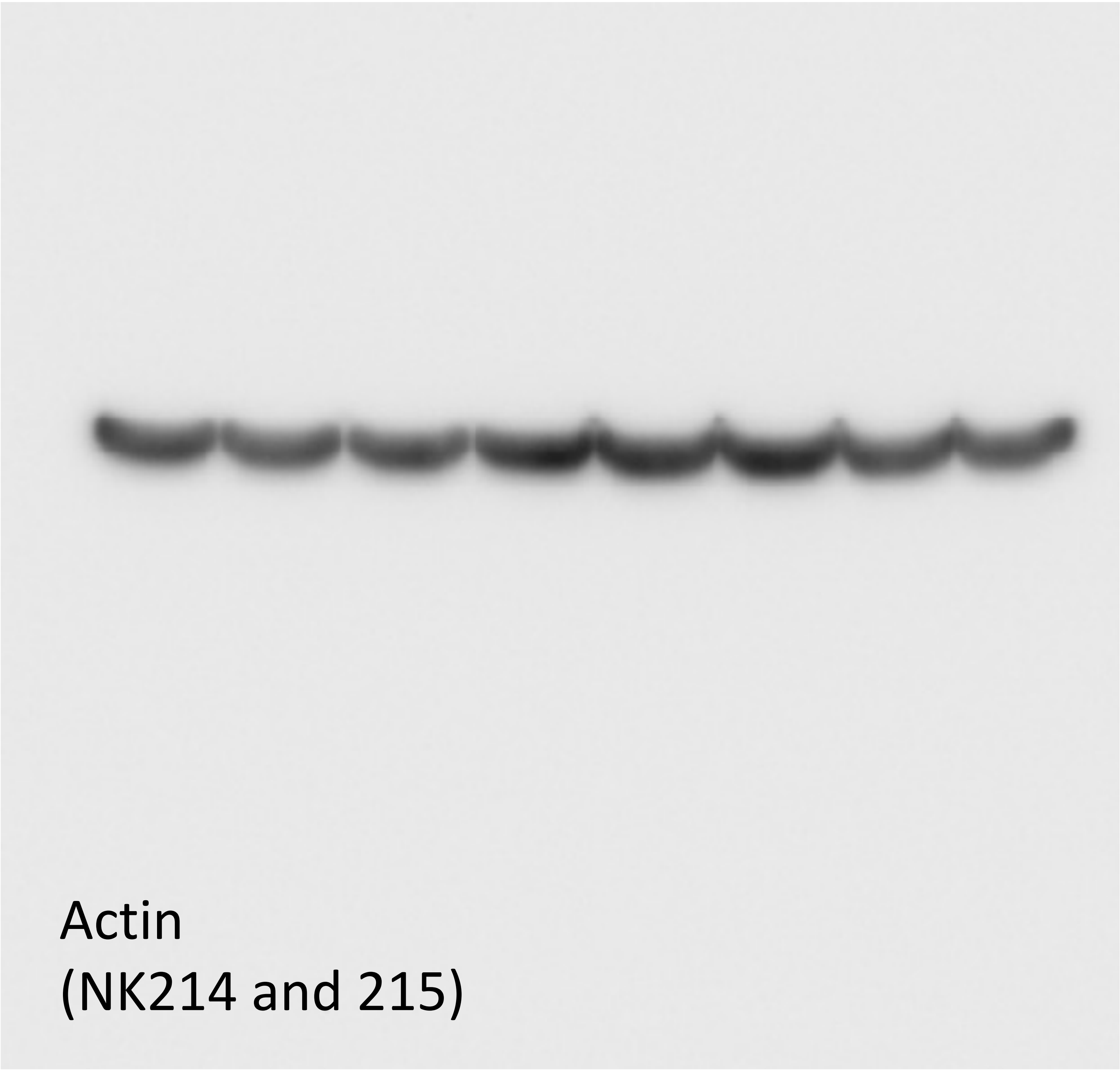
Actin
(NK214 and 215)

**Supplementary Table 1**

| | PTP1B DQ/ JAK1 (PDB ID: 8EXJ) | PTP1B DQ/ JAK2 (PDB ID: 8EXK) | PTP1B DQ/ JAK3 (PDB ID: 8EXM) | PTP1B DQ/ TYK2 (PDB ID: 8EXN) | PTP1B DQ apo (PDB ID: 8EXI) |
|---|---|---|---|---|---|
| **Data Collection** | | | | | |
| Space group | P $4_3$ $2_1$ 2 | P $4_3$ $2_1$ 2 | P $4_3$ $2_1$ 2 | P $4_3$ $2_1$ 2 | P $3_1$ 2 1 |
| Cell dimensions<br>a, b, c (Å)<br>α, β, γ (°) | 88.57 88.57 132.43<br>90 90 90 | 88.48 88.48 130.41<br>90 90 90 | 88.59 88.59 132.16<br>90 90 90 | 88.32 88.32 131.23<br>90 90 90 | 88.72 88.72 104.65<br>90 90 120 |
| $R_{meas}$ (%) | 16.12 (471)* | 5.93 (84.21) | 5.36 (56.53) | 9.82 (318)* | 4.89 (121.8) |
| I / sigma(I) | 9.56 (0.27) | 21.46 (2.14) | 27.61 (3.21) | 11.38 (0.76) | 23.89 (1.84) |
| $CC_{1/2}$ (%) | 99.8 (11)* | 99.9 (83) | 100 (91) | 99.8 (27)* | 100 (67.7) |
| Completeness (%) | 97.33 (73.58) | 99.77 (98.72) | 99.49 (95.84) | 99.54 (97.94) | 99.87 (98.88) |
| Redundancy | 8.3 (5.2) | 7.4 (7.2) | 11.4 (8.4) | 7.3 (6.4) | 9.0 (9.1) |
| **Refinement** | | | | | |
| Resolution (Å) | 45.5 - 2.30 (2.38 - 2.30) | 44.25 - 2.09 (2.17 - 2.09) | 39.62 - 2.35 (2.43 - 2.35) | 45.24 - 2.15 (2.23 - 2.15) | 44.36 - 1.59 (1.65 - 1.59) |
| No. reflections | 23449 (1743) | 26961 (2623) | 22507 (2118) | 28847 (2809) | 63292 (6181) |
| R-work (%)** | 20.66 (41.52) | 21.00 (28.36) | 20.61 (26.29) | 21.69 (38.05) | 19.05 (27.36) |
| R-free (%)** | 23.86 (43.63) | 25.02 (30.49) | 24.76 (30.08) | 24.04 (38.13) | 21.20 (28.79) |
| **No. atoms** | | | | | |
| protein | 2426 | 2412 | 2393 | 2408 | 2412 |
| ligand | 21 | 5 | 13 | 21 | 12 |
| solvent | 53 | 70 | 86 | 74 | 162 |
| **B factors** | | | | | |
| protein | 69.06 | 51.44 | 61.62 | 64.31 | 31.28 |
| ligand | 81.12 | 54.30 | 64.76 | 72.21 | 48.49 |
| solvent | 62.18 | 47.33 | 58.01 | 61.04 | 38.37 |
| **r.m.s. deviations** | | | | | |
| RMS(bonds) | 0.008 | 0.007 | 0.007 | 0.006 | 0.006 |
| RMS(angles) | 0.89 | 0.83 | 0.78 | 0.79 | 0.83 |
| Ramachandran favored (%) | 93.36 | 95.64 | 92.62 | 94.24 | 97.64 |
| Ramachandran allowed (%) | 6.31 | 3.36 | 7.05 | 5.42 | 2.03 |
| Ramachandran outliers (%) | 0.33 | 1.01 | 0.34 | 0.34 | 0.34 |
| Rotamer outliers (%) | 4.28 | 1.95 | 2.78 | 5.04 | 1.16 |

| | PTP1B DQC/ JAK2 pYpY (PDB ID: 8EYB) | PTP1B DQC/ TYK2 pYpY (PDB ID: 8EYC) | PTP1B DQC/ JAK2 pYY (PDB ID: 8EYA) | PTP1B DQC/ JAK2 YpY (PDB ID: 8F88) |
|---|---|---|---|---|
| **Data Collection** | | | | |
| Space group | P $3_2$ 2 1 | P $2_1$ $2_1$ $2_1$ | P $3_1$ 2 1 | P $2_1$ |
| Cell dimensions a, b, c (Å) α, β, γ (°) | 88.83 88.83 197.45 90 90 120 | 44.48 88.22 97.66 90 90 90 | 88.38 88.38 190.33 90 90 120 | 88.22 176.23 43.79 90 90.29 90 |
| $R_{meas}$ (%) | 6.05 (73.42) | 16.08 (245.6)* | 6.01 (74.27) | 8.86 (79.95) |
| I /sigma(I) | 16.67 (2.58) | 10.44 (0.91) | 18.94 (2.51) | 12.86 (1.69) |
| $CC_{1/2}$ (%) | 99.9 (85) | 99.7 (37.7)* | 99.9 (77) | 99.8 (74) |
| Completeness (%) | 99.67 (99.26) | 99.56 (97.73) | 99.63 (99.25) | 99.05 (95.38) |
| Redundancy | 5.0 (5.2) | 6.5 (6.3) | 5.6 (5.6) | 3.5 (3.4) |
| **Refinement** | | | | |
| Resolution (Å) | 43.33 - 2.09 (2.17 - 2.01) | 48.84 - 2.79 (2.89 - 2.79) | 48.85 - 2.34 (2.43 - 2.34) | 42.79 - 3.09 (3.20 - 3.09) |
| No. reflections | 53432 (5267) | 64878 (6043) | 36591 (3580) | 24126 (2290) |
| R-work | 21.70 (33.12) | 22.16 (38.84) | 20.62 (29.57) | 25.59 (33.46) |
| R-free | 25.85 (40.68) | 27.00 (46.36) | 24.16 (32.79) | 28.72 (38.25) |
| **No. atoms** | | | | |
| protein | 4914 | 2364 | 4792 | 6873 |
| ligand | 23 | - | 16 | 24 |
| solvent | 175 | 7 | 118 | 37 |
| **B factors** | | | | |
| protein | 53.65 | 83.32 | 58.77 | 62.73 |
| ligand | 62.88 | - | 66.10 | 76.59 |
| solvent | 52.48 | 71.11 | 53.37 | 60.63 |
| r.m.s. deviations | | | | |
| RMS(bonds) | 0.006 | 0.005 | 0.007 | 0.009 |
| RMS(angles) | 0.76 | 0.75 | 0.81 | 1.27 |
| Ramachandran favored (%) | 95.81 | 95.61 | 96.61 | 94.43 |
| Ramachandran allowed (%) | 3.69 | 4.05 | 2.71 | 4.44 |
| Ramachandran outliers (%) | 0.50 | 0.34 | 0.68 | 1.14 |
| Rotamer outliers (%) | 1.17 | 1.69 | 1.14 | 8.58 |

Values in parentheses are for highest-resolution shell. *Criteria for data cut-off were from (Karplus and Diederichs, 2012) using the split-half correlation coefficient ($CC_{1/2}$). ** Data are for structures where second tyrosine occupies the catalytic pocket.
