# Supplementary material for: Structure guided studies of the interaction between PTP1B and JAK
Source: Commun Biol. 2023 Jun 14;6:641. doi: 10.1038/s42003-023-05020-9 (PMC10267100; doi:10.1038/s42003-023-05020-9)
Supplement: Supplementary file 3 — Description of Additional Supplementary Files [file 42003_2023_5020_MOESM3_ESM.pdf]

Description of Additional Supplementary Files

File name: Supplementary Data 1
Description: Raw data from NMR dephosphorylation assays.

File name: Supplementary Data 2
Description: Raw data from incucyte assays.

File name: Supplementary Data 3
Description: Raw data from malachite green assays on various pepeitdes.

File name: Supplementary Data 4
Description: Raw data from NMR experiments looking at dephosphorylation rates.

File name: Supplementary Data 5
Description: Raw data from M1 dephosphorylation assay

File name: Supplementary Data 6
Description: Raw data from SPR direct binding curves

File name: Supplementary Data 7
Description: Raw data from thermal shift assays with peptides and PTP1B.
