# Supplementary material for: Structure guided studies of the interaction between PTP1B and JAK
Source: Commun Biol. 2023 Jun 14;6:641. doi: 10.1038/s42003-023-05020-9 (PMC10267100; doi:10.1038/s42003-023-05020-9)
Supplement: Supplementary file 11 — Reporting Summary [file 42003_2023_5020_MOESM11_ESM.pdf]

nature portfolio

Corresponding author(s): Jeffrey J. Babon

Last updated by author(s): Jun 2, 2023

Reporting Summary

Nature Portfolio wishes to improve the reproducibility of the work that we publish. This form provides structure for consistency and transparency in reporting. For further information on Nature Portfolio policies, see our Editorial Policies and the Editorial Policy Checklist.

Statistics

For all statistical analyses, confirm that the following items are present in the figure legend, table legend, main text, or Methods section.

n/a	Confirmed	
☐	☒	The exact sample size (*n*) for each experimental group/condition, given as a discrete number and unit of measurement
☐	☒	A statement on whether measurements were taken from distinct samples or whether the same sample was measured repeatedly
☒	☐	The statistical test(s) used AND whether they are one- or two-sided<br>*Only common tests should be described solely by name; describe more complex techniques in the Methods section.*
☒	☐	A description of all covariates tested
☒	☐	A description of any assumptions or corrections, such as tests of normality and adjustment for multiple comparisons
☐	☒	A full description of the statistical parameters including central tendency (e.g. means) or other basic estimates (e.g. regression coefficient) AND variation (e.g. standard deviation) or associated estimates of uncertainty (e.g. confidence intervals)
☒	☐	For null hypothesis testing, the test statistic (e.g. *F*, *t*, *r*) with confidence intervals, effect sizes, degrees of freedom and *P* value noted<br>*Give P values as exact values whenever suitable.*
☒	☐	For Bayesian analysis, information on the choice of priors and Markov chain Monte Carlo settings
☒	☐	For hierarchical and complex designs, identification of the appropriate level for tests and full reporting of outcomes
☒	☐	Estimates of effect sizes (e.g. Cohen's *d*, Pearson's *r*), indicating how they were calculated

Our web collection on statistics for biologists contains articles on many of the points above.

Software and code

Policy information about availability of computer code

Data collection	BIAcore experiments were collected with the help of BIAcore control software.<br>Stability assay data were collected using a Tycho NT.6 Nonotempter.<br>Incucyte assay data was collected using a Sartorius IncuCyte SX5.
Data analysis	X-ray crystallography data were processed using XDS (Version Nov 1, 2016; BUILT = 2016120S(. Phenix 1.19.2, Phaser-MR was used for molecular replacement and phenix.refine was used for refinement. Coot version 0.9 was used for model building along with Coot as part of CCP2 version 7.1.<br>BIAcore experiments were evaluated using BIAcore insight evaluation software and Graphpad Prism 9.0 was used o further analyze<br>Stability assays were collected and analysed using a Tycho NT.6 Nanotemper. Graphpad prism 9.0 was used to further analyse stability assay data.<br>Incucyte assay data was analysed using Essenbiosciences Incucyte software.

For manuscripts utilizing custom algorithms or software that are central to the research but not yet described in published literature, software must be made available to editors and reviewers. We strongly encourage code deposition in a community repository (e.g. GitHub). See the Nature Portfolio guidelines for submitting code & software for further information.

Data

Policy information about availability of data

All manuscripts must include a data availability statement. This statement should provide the following information, where applicable:

- Accession codes, unique identifiers, or web links for publicly available datasets
- A description of any restrictions on data availability
- For clinical datasets or third party data, please ensure that the statement adheres to our policy

Atomic coordinates for all PTP1B structures have been deposited in the Protein Data Bank with the accession numbers 8EXJ, 8EXK, 8EXM, 8EXN, 8EXI, 8EYB, 8EYC, 8EYA, 8F88.
The data used to generate Figure 1, Figure 2 and Figure 4 have been made available in the source data files.

Field-specific reporting

Please select the one below that is the best fit for your research. If you are not sure, read the appropriate sections before making your selection.

☒ Life sciences ☐ Behavioural & social sciences ☐ Ecological, evolutionary & environmental sciences

For a reference copy of the document with all sections, see nature.com/documents/nr-reporting-summary-flat.pdf

Life sciences study design

All studies must disclose on these points even when the disclosure is negative.

Sample size	Sample size for each experiment was determined by minimum number required to achieve similar robust results, for many experiments, only a single technical replicate was required.. The M1 dose response assay with phosphatases was performed once, the incucyte assay was performed independently 3 times. Western blots were repeated between 3 and 4 times independently. SPR assays were performed once or twice, and stability assays were performed independently twice.
Data exclusions	Some data points were excluded from BIAcore and stability assays due to technical errors such as a damaged flow cell, or no melting curves detected. These datasets or points were excluded from analysis.
Replication	Incucyte assays were performed independently 3 times. Western blots were repeated independently at least 3 times. Biacore experiments were performed up to 5 times and stability assays were performed independently twice. malachite green assays were performed individually 3 times, and NMR experiments once or twice. The spread of data is shown throughout the paper through use of individual data points and SD or SEM error bars.
Randomization	Randomization was not relevant to most of our study as experiments were quantitative and not qualitative - Our studies looked to understand to the interaction of PTP1B with the JAK proteins, and as such, mitigation of human bias was done by collection of datapoints using computer software.
Blinding	Figure 1A was blinded to the person who collected the data and only unblinded upon data analysis. Blinding was not relevant to the remainder of experiments.

Reporting for specific materials, systems and methods

We require information from authors about some types of materials, experimental systems and methods used in many studies. Here, indicate whether each material, system or method listed is relevant to your study. If you are not sure if a list item applies to your research, read the appropriate section before selecting a response.

Materials & experimental systems

n/a	Involved in the study	
☐	☒	Antibodies
☐	☒	Eukaryotic cell lines
☒	☐	Palaeontology and archaeology
☒	☐	Animals and other organisms
☒	☐	Human research participants
☒	☐	Clinical data
☒	☐	Dual use research of concern

Methods

n/a	Involved in the study	
☒	☐	ChIP-seq
☒	☐	Flow cytometry
☒	☐	MRI-based neuroimaging

Antibodies

Antibodies used	p-JAK1 (44-422G), FLAG (WEHI MAB 9H1), HALO (G9211), PTP1B (ab124375), Actin (Sc-47778), anti-rabbit HRP (NA934), anti-mouse HRP (NA931), and anti-rat HRP (31470). Full details of antibodies, species, supplier and dilution is listed in the methods.
Validation	p-JAK1 (44-422G), The antiserum was produced against a chemically synthesized phosphopeptide derived from a region of human JAK1 protein that contains tyrosines 1022 and 1023. The sequence is conserved in mouse and pig. Publication using this antibody: Kiessling, Stephan, et al. "Functional expression of the interleukin-11 receptor α-chain and evidence of antiapoptotic effects in human colonic epithelial cells." Journal of Biological Chemistry 279.11 (2004): 10304-10315.<br>FLAG (WEHI MAB 9H1), validated against recombinant protein in-house at WEHI.<br>HALO (G9211), Validated by Promega by FACS. Publication using antibody: Gandin, Valentina, et al. "Cap-dependent translation initiation monitored in living cells." Nature communications 13.1 (2022): 1-15.<br>PTP1B (ab124375), validated by western blot and FACS using WB: PTP1B-transfected HEK293T, HepG2, HeLa, COS7, Jurkat, MDCK, PC12, and MCF7 cell lysates IHC-P: Human colon, Human lymph node and Human lymphoma tissuesICC/IF: COS7 cells transiently transfected by PTP1B. Flow Cyt (Intra): PTP1B-transfected HEK293T cells<br>Actin (Sc-47778), validated by blotting and immunohistochemistry. 1. Teicher, M.H. and Barber, N.I. 1990. COSIFIT: an interactive program for simultaneous multioscillator cosinor analysis of time-series data. Comput. Biomed. Res. 23: 283-295.<br>2. Zou, Z., et al. 2021. MAP4K4 induces early blood-brain barrier damage in a murine subarachnoid hemorrhage model. Neural. Regen. Res. 16:<br>325-332.

Eukaryotic cell lines

Policy information about cell lines

Cell line source(s)	M1 cell lines from WEHI and HEK293 cells sourced originally from ATCC.
Authentication	Cell lines were not genetically authenticated, however their morphology was visually confirmed.
Mycoplasma contamination	Invivogen PlasmiTest kit was used to ensure cell lines were mycoplasma free.
Commonly misidentified lines (See ICLAC register)	No commonly misidentified cell lines were used in this study.
